# Molecular phylogeny of the diatom genus *Planothidium* with the description of *Paraplanothidium* gen. nov. and *Pseudoplanothidium* gen. nov.

**DOI:** 10.1371/journal.pone.0333782

**Published:** 2025-11-06

**Authors:** Natalia Tseplik, Yevhen Maltsev, Anton Glushchenko, Sergei Genkal, Maxim Kulikovskiy

**Affiliations:** 1 K.A. Timiryazev Institute of Plant Physiology RAS, Moscow, Russia; 2 Papanin Institute for Biology of Inland Waters, Russian Academy of Sciences, Borok, Russia; CEA lRlG: Commissariat a l'energie atomique et aux energies alternatives lnstitut de Recherche Interdisciplinaire de Grenoble, FRANCE

## Abstract

One of the characteristic features of the monoraphid genus *Planothidium* is the structure of the central part of the rapheless valve. *Planothidium* is divided into three groups based on this feature: with a sinus, a cavum, or with uninterrupted striae. Representatives of all three groups of *Planothidium* from the Kamchatka Peninsula have been studied using molecular (genetic markers 18S rDNA and *rbc*L) and morphological approach. Two new genera are separated from *Planothidium*: *Paraplanothidium* gen. nov., characterised by a cavum, and *Pseudoplanothidium* gen. nov., characterised by the absence of a horseshoe-shaped depression on the rapheless valve; this decision is supported by molecular data. Three new species from the new genera are described based on light and scanning electron microscopy as well as molecular analysis: *Paraplanothidium laevis*, *Pseudoplanothidium foliiformis* and *Pseudoplanothidium minutum*. New taxonomic combinations are proposed for previously described *Planothidium* species. This study contributes to the research of monoraphid diatoms taxonomy.

## Introduction

The taxonomy of monoraphid diatoms has gone through a lot of changes in the recent decades. Historically, most of diatom species with only one raphe were included in the genus *Achnanthes* Bory, which was a very heterogeneous taxon that included several hundreds of species [1]. In the 1990s, revisions of this group were initiated in order to avoid confusion and complications. One of the first researchers to work in this direction were F. Round and L. Bukhtiyarova, who separated five genera from *Achnanthes* [2,3], including *Planothidium* Round et Bukhtiyarova. *Planothidium* was established to include *Achnanthes lanceolata* (Brébisson ex Kützing) Grunow and closely related species, with *A. lanceolata* designated as the type species [3]. It was distinguished by more or less flat valves, multiseriate striae on both valves, long terminal raphe fissures that are curved to one side and a horseshoe-shaped structure on the one side of the rapheless valve; concerning the last feature, however, the authors note that the *Planothidium delicatulum* (Kützing) Round et Bukhtiyarova species group has uninterrupted striae in the central area (i.e., no horseshoe-shaped structure).

Later, Lange-Bertalot [4] suggested the genus *Achnantheiopsis* Lange-Bertalot that included 38 monoraphid species that could not be assigned to either *Achnanthes* s. str. or *Achnanthidium* Kützing, nor did they fit the descriptions of *Eucocconeis* Cleve ex F.Meister and *Psammothidium* Bukhtiyarova & Round. *Achnantheiopsis* was differentiated from other genera by a complex of features, such as valve outline, the structure of central and distal raphe ends, multiseriate striae, internally thickened interstriae, and the ultrastructure of areolae. However, this name turned out to be a synonym of *Planothidium*, since these genera were based on the same type species *A. lanceolata*.

The horseshoe-shaped structure (German: hufeisenförmiger Fleck) observed in some species assigned to *Planothidium* was shown to be differentiated into two types: a single or a double depression, termed sinus and cavum respectively [5]. A sinus is characteristic for the *Planothidium lanceolatum* (Brébisson ex Kützing) Lange-Bertalot species group, while a cavum is present in *Planothidium frequentissimum* (Lange-Bertalot) Lange-Bertalot and allied taxa. Later, the importance of this feature for the genus was discussed [6] and four groups of *Planothidium* species were suggested based on the structure of the rapheless valve central area: with a sinus, with a cavum, with uninterrupted striae on the rapheless valve or with an asymmetric hyaline central area (without a horsehoe-shaped structure of either type).

In recent years, the type materials of some previously established *Planothidium* species were investigated in order to enhance our understanding of the taxonomy of this group. This included the generitype *P. lanceolatum* (which was lectotypified as well) [7], *P. frequentissimum* [8] and *P. delicatulum* [9].

Morphological and molecular methods were used to investigate strains of several *Planothidium* species (including *P. lanceolatum* and *P. frequentissimum*) [10]. The phylogenetic analysis recovered three distinct groups of species: one group characterised by a cavum, one by a sinus and the last one without either type of horseshoe-shaped structure. Thus the taxonomic significance of this feature was established. As an additional distinguishing characteristic, the authors note that the *P. lanceolatum* s.l. clade has striae that consist of 2–3 rows of areolae with smaller areolae in the middle row, while the *P. frequentissimum* s.l. clade has 3–4 rows of areolae in a stria and the areolae are all similarly sized. As another result of this study several new species were described from all three groups of *Planothidium*, namely *P. cryptolanceolatum* Jahn et Abarca, *P. taeansa* Jahn et Abarca, *P. naradoense* Jahn et Zimmermann and *P. suncheonmanense* Jahn et Zimmermann [10]. For *P. victori* Novis, Braidwood et Kilroy, its conspecificity with *P. caputium* Zimmermann et Jahn was established and an epitype strain was designated [10].

While a sinus is characteristic only for *Planothidium*, several other monoraphid genera possess a cavum. These are *Skabitschewskia* Kulikovskiy et Lange-Bertalot, *Gliwiczia* Kulikovskiy, Lange-Bertalot et Witkowski, *Planoplatessa* Kulikovskiy, Glushchenko et Kociolek and *Xenobennettella* Witkowski et Riaux-Gobin. These genera are clearly differentiated from each other by a complex of morphological features, despite having a similarity in the stucture of the central part of the rapheless valve. Furthermore, *Gliwiczia* has a cavum on both valves and not just the rapheless valve, suggesting that this structure is not homologous with the cavum in *Planothidium* [10]. This leads to the idea that the cavum as a morphological feature arose several times in monoraphid diatoms.

This work contains a molecular and morphological analysis of 22 *Planothidium* strains from the Kamchatka Peninsula, Russia. The division of *Planothidium* into three groups based on the structure of the rapheless valve central area is shown. A more narrow understanding of the genus *Planothidium* is suggested based on these findings and two new genera are described to include species with a cavum and without any horseshoe-shaped structure on the rapheless valve. Three new species are described as well.

## Materials and methods

*Sample collection*. For this study we used samples collected in July 2021 on the Kamchatka Peninsula, Russia. Samples were collected on publicly available territories. Detailed information on used strains is given in Table 1.

**Table 1:** List of strains used in this study.

Strain	Sampling locality	Collection date	Sample type	Coordinates	Sample no.
*Paraplanothidium laevis* gen. et sp. nov. CBMCkam113	Lake Kurazhechnoye	13 July 2021	detritus and scrapes from stones	N 56.365.238 E 160.894.019	84
*Paraplanothidium laevis* gen. et sp. nov. CBMCkam115	Lake Kurazhechnoye	13 July 2021	detritus and scrapes from stones	N 56.365.238 E 160.894.019	84
*Paraplanothidium laevis* gen. et sp. nov. CBMCkam128	Lake Kurazhechnoye	13 July 2021	detritus and scrapes from stones	N 56.365.238 E 160.894.019	84
*Planothidium piipii* kam197	mineral creek near Pushchinskiye mineral springs	11 July 2021	scrapes from stones	N 54.01.616 E 158.040.972	44
*Planothidium piipii* kam233	Lake Kurazhechnoye	13 July 2021	sand and detritus	N 56.365.238 E 160.894.019	82
*Paraplanothidium laevis* gen. et sp. nov. CBMCkam265	Verkhne-Paratunskiy hot spring	18 July 2021	detritus	N 52.823.669 E 158.163.369	180
*Planothidium piipii* kam277	Pravaya Kamchatka river	15 July 2021	detritus	N 54.024.019 E 157.849.01	121
*Paraplanothidium tyushovii* comb. nov. kam288	Greshnaya river	15 July 2021	sand and detritus	N 55.923.778 E 158.701.194	116
*Planothidium novograblenovii* kam295	Verkhniy Paratunskiy hot sping	18 July 2021	detritus	N 52.823.669 E 158.163.369	186
*Paraplanothidium laevis* gen. et sp. nov. CBMCkam297	Greshnaya river	15 July 2021	detritus	N 55.923.778 E 158.701.194	118
*Paraplanothidium laevis* gen. et sp. nov. CBMCkam298	Greshnaya river	15 July 2021	detritus	N 55.923.778 E 158.701.194	118
*Paraplanothidium laevis* gen. et sp. nov. CBMCkam300	Greshnaya river	15 July 2021	detritus	N 55.923.778 E 158.701.194	118
*Planothidium piipii* kam303	mineral creek near Pushchinskiye mineral springs	11 July 2021	scrapes from stones	N 54.01.616 E 158.040.972	44
*Planothidium piipii* kam316	Lake Kurazhechnoye	13 July 2021	detritus and scrapes from stones	N 56.365.238 E 160.894.019	84
*Paraplanothidium laevis* gen. et sp. nov. CBMCkam322	Verkhne-Paratunskiy hot spring	18 July 2021	detritus	N 52.823.669 E 158.163.369	186
*Paraplanothidium dyakonovii* comb. nov. kam373	Dachnye hot springs	18 July 2021	detritus, sand and clay	N 52.530.139 E 158.191.755	219
*Planothidium piipii* kam407	Bolshaya Kimitina river	12 July 2021	sand and detritus	N 55.042.225 E 158.883.171	69
*Pseudoplanothidium foliiformis* gen. et sp. nov. CBMCkam430	Yuzhnaya Bay	17 July 2021	phytoplankton	N 52.952.434 E 158.687.17	166
*Pseudoplanothidium foliiformis* gen. et sp. nov. CBMCkam446	Yuzhnaya Bay	17 July 2021	phytoplankton	N 52.952.434 E 158.687.17	166
*Pseudoplanothidium foliiformis* gen. et sp. nov. CBMCkam470	Yuzhnaya Bay	17 July 2021	benthos and detritus	N 52.952.434 E 158.687.17	167
*Pseudoplanothidium minutum* gen. et sp. nov. CBMCkam478	Yuzhnaya Bay	17 July 2021	phytoplankton	N 52.952.434 E 158.687.17	166
*Pseudoplanothidium foliiformis* gen. et sp. nov. CBMCkam483	Yuzhnaya Bay	17 July 2021	phytoplankton	N 52.952.434 E 158.687.17	166

*Culturing.* A part from each sample was transferred to the WC liquid culturing medium [11]. Single cells were micropipetted under an inverted microscope Axio Vert. A1 (Zeiss, Oberkochen, Germany) in order to establish monoclonal strains. Non-axenic unialgal cultures were maintained in the WC culturing medium at 22–25°C in a growth chamber with a 12:12 h light:dark photoperiod for approximately 1 month before performing microscopical investigation and DNA extraction. The strains were deposited in the Culture and Barcode Collection of Microalgae and Cyanobacteria “Algabank” (CBМC) at K.A. Timiryazev Institute of Plant Physiology RAS.

*Slide preparation*. The samples were boiled in concentrated hydrogen peroxide (≈37%) to dissolve organic matter. The samples were then rinsed four times with deionized water every 12 hours. After decanting and rinsing with up to 100 mL of deionized water, the suspension was put onto coverslips and left to dry at room temperature. Permanent diatom slides were mounted in Naphrax^®^. Light microscopic (LM) observations were performed with a Zeiss Scope A1 microscope equipped with an oil immersion objective (100 × , n.a. 1.4, Nomarski differential interference contrast [DIC]) and Zeiss AxioCam ERc 5s camera. For scanning electron microscopy (SEM), parts of the suspensions were fixed on aluminum stubs after air-drying. The stubs were sputter coated with 50 nm of gold. Valve ultrastructure was examined by means of a JSM-6510LV scanning electron microscope (Institute for Biology of Inland Waters RAS, Borok, Russia).

*Molecular study*. Genomic DNA of the studied diatom strains was extracted from fresh cultures by Chelex 100 Chelating Resin (Bio-Rad Laboratories, Hercules, CA, USA) using protocol 2.2. Nuclear gene 18S rDNA and plastid *rbc*L gene were amplified. For the highly variable V4 region of 18S rDNA (404–413 bp) D512for and D978rev primers were used [12]. The plastid *rbc*L (915–954 bp) was amplified using *rbc*L404+ and rbcL1444- [13] primers.

PCR amplifications were performed using premade mastermixes (ScreenMix, Evrogen, Moscow, Russia). The amplification of the 18S rDNA was performed using the following program: 5 min of denaturation at 95°C; followed by 35 cycles of denaturation at 94°C (30 s), annealing at 52°C (30 s), and elongation at 72°C (50 s); with a final extension at 72°C (7 min), subsequently held at 12°C. The amplification of the *rbc*L gene was performed using the following program: 4 min of denaturation at 94°C; followed by 44 cycles of denaturation at 94°C (50 s), annealing at 53°C (50 s), and elongation at 72°C (80 s); with a final extension at 72°C (10 min), subsequently held at 12°C.

The PCR products were sized on a 1.0% agarose gel stained with SYBR^TM^ Safe (Life Technologies, Carlsbad, CA, USA) and then purified using a mixture of FastAP, 10 × FastAP Buffer, Exonuclease I (Thermo Fisher Scientific, Waltham, MA, USA), and water. The purified PCR products were sequenced by Sanger Sequencing method using a Genetic Analyzer 3500 instrument (Applied Biosystems, Waltham, MA, USA).

**Fig 1:**
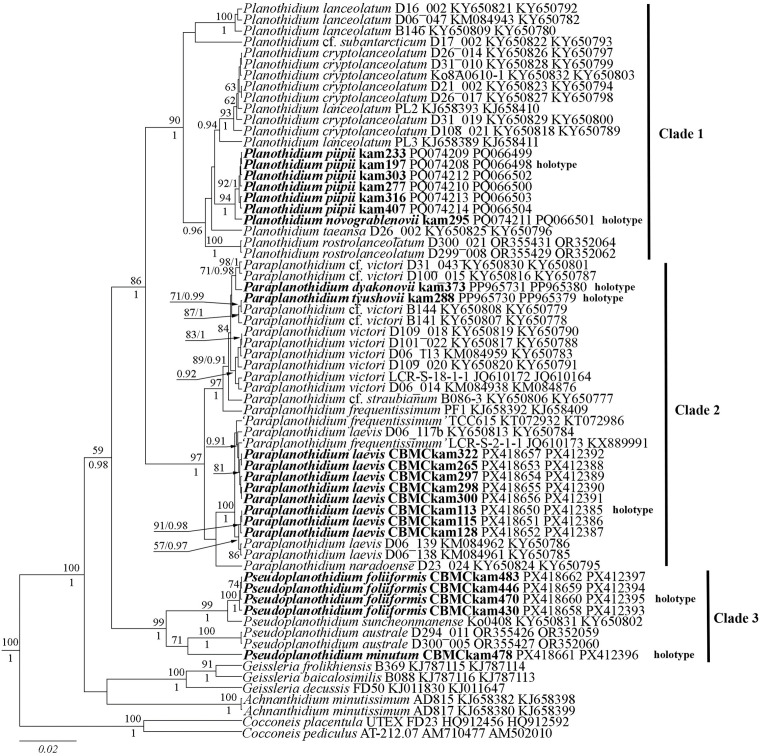
Phylogenetic position of the new *Paraplanothidium* and *Pseudoplanothidium* species (indicated in bold) based on Bayesian inference for the partial *rbc*L and 18S rRNA genes. The total length of the alignment is 1,318 characters. Bootstrap supports (BS) from ML (constructed by RAxML) and posterior probabilities (PP) from BI (constructed by Beast) are presented on the nodes in order. Only BS and PP above 50 and 0.9 are shown. Strain numbers (if available) and GenBank numbers are indicated for all sequences.

Newly obtained sequences were manually edited in Ridom TraceEdit ver. 1.1.0 (Ridom GmbH, Münster, Germany) and Mega ver. 7 software [14]. The reads were combined with GenBank-extracted sequences of 49 diatom species and two out-group *Cocconeis* Ehrenberg species (taxa names and Accession Numbers are given in Fig 1). The 18S rDNA and *rbc*L sequences were aligned separately using the G-INS-i algorithm in the Mafft ver. 7 software (RIMD, Osaka, Japan) [15]. The resulting data set comprised of 403 and 915 nucleotide sites for nuclear 18S rDNA, and plastid *rbc*L regions, respectively. After removal of the unpaired regions, the aligned 18S rRNA gene sequences were combined with the *rbc*L gene sequences into a single matrix for concatenated 18S rDNA and *rbc*L tree.

The Bayesian inference (BI) method was performed to infer the phylogenetic position of new diatom strains using Beast ver. 1.10.1 software (BEAST Developers, Auckland, New Zealand) [16]. The most appropriate partition-specific substitution models, shape parameter α and a proportion of invariable sites (pinvar) were recognized by the Bayesian information criterion (BIC) in jModelTest ver. 2.1.10 software (Vigo, Spain) [17]. This BIC-based model selection procedure selected the following models, shape parameter α and a proportion of invariable sites (pinvar): TrN + I + G, α = 0.3260 and pinvar = 0.6270 for 18S rDNA; HKY + I, pinvar = 0.8240 for the first codon position of the *rbc*L gene; TPM1 + I, pinvar = 0.8570 for the second codon position of the *rbc*L gene; HKY + I + G, α = 0.8820 and pinvar = 0.0750 for the third codon position of the *rbc*L gene. However, the HKY model was applied instead of TrN and TPM1, as the most similar applicable options for BI. A speciation model was performed by a Yule process tree prior. Five MCMC analyses were run for 8 million generations (burn-in 1,000 million generations). The convergence diagnostics was performed in the Tracer ver. 1.7.1 software (MCMC Trace Analysis Tool, Edinburgh, United Kingdom) [16]. The initial 15% trees were removed, the rest retained to construct a final chronogram with 90% posterior probabilities. The robustness of tree topologies was assessed by boot-strapping the data set with Maximum Likelihood (ML) analysis using RAxML software [18]. The ML bootstrapping was performed with 1,000 replicas. Trees were viewed and edited using FigTree ver. 1.4.4 (University of Edinburgh, Edinburgh, United Kingdom) and Adobe Photoshop CC ver. 19.0 software.

The 18S rDNA and *rbc*L sequences were also used to estimate the degree of similarity between gene sequences of different *Planothidium* strains. Using Mega7 software, the p-distances were determined to calculate the sequence similarity with the formula (1–p) × 100.

*Nomenclature*. The electronic version of this article in Portable Document Format (PDF) in a work with an ISSN or ISBN will represent a published work according to the International Code of Nomenclature for algae, fungi, and plants, and hence the new names contained in the electronic publication of a PLOS One article are effectively published under that Code from the electronic edition alone, so there is no longer any need to provide printed copies.

The online version of this work is archived and available from the following digital repositories: PubMed Central, LOCKSS.

## Results

On the phylogenetic tree, species of *Planothidium* sensu lato are divided into three distinct clades (Fig 1). Clade 1 includes strains of species that possess a sinus on the rapheless valve. Clade 2 contains taxa characterised by a cavum and is further divided into two subclades. Lastly, Clade 3 includes species that are characterised by an absence of any horseshoe-shaped structure in the central part of the rapheless valve.

Since the morphological differences between these groups of species are clear and well-supported by the molecular data, we propose two new genera to be separated from *Planothidium* based on the structure of the central area on the rapheless valve.

The terminology in species descriptions and discussion follows [10,19,20].

### *Planothidium* Round et Bukhtiyarova s. str. (Figs 2–4)

**Emended description. LM**. Frustules monoraphid. Valves elliptic to elliptic-lanceolate, with variably shaped ends. Raphe straight, filiform, with straight expanded central ends and distal ends that are curved to one side. Axial and central areas variable; a sinus (uncovered depression) is present on one side of the central area on the rapheless valve.

**Fig 2:**
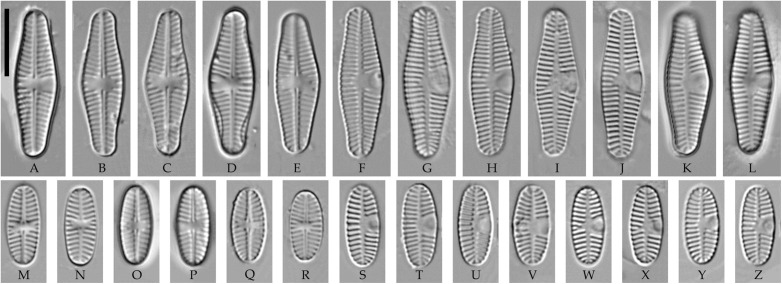
*Planothidium piipii* Tseplik, Glushchenko, Maltsev et Kulikovskiy. (A–L) Strain kam197, slide 08029, type material; (M–Z) strain kam303, slide 08135. Light microscopy, differential interference contrast. (A–E, M–R) raphe valves; (F–L, S–Z) rapheless valves. Scale bar 10 μm.

**Fig 3:**
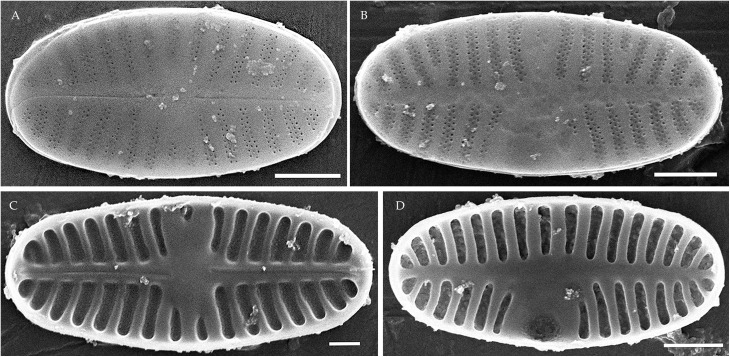
Planothidium piipii. Strain kam303. Scanning electron microscopy. (A) raphe valve, external view; (B) rapheless valve, external view; (C) raphe valve, internal view; (D) rapheless valve, internal view. Scale bar 2 μm (A, B), 1 μm (C, D).

**Fig 4:**
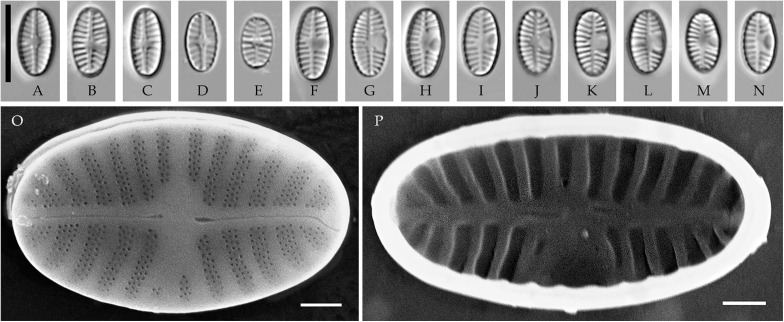
*Planothidium novograblenovii* Tseplik, Glushchenko, Maltsev et Kulikovskiy. Strain kam295, slide 08127, type material. (A–N) Light microscopy, differential interference contrast. (A–E) raphe valves; (F–N) rapheless valves. Scale bar 10 μm. (O, P) Scanning electron microscopy. (O) raphe valve, external view; (P) rapheless valve, internal view. Scale bar 1 μm.

**SEM**. External central raphe ends straight, expanded, distal ends turned to one side and extended onto the mantle. Internal central raphe ends slightly bent to opposite sides, distal ends terminate in small helictoglossae. Striae on both valves bi- to multiseriate, each consisting of up to 5 rows of small circular areolae that are closed on the inside by a hymen. Internally, the interstriae are distinctly raised.

### *Paraplanothidium* Tseplik, Glushchenko, Genkal, Maltsev et Kulikovskiy gen. nov. (Figs 5–7).

**Type species (here designated)**: *Paraplanothidium laevis* sp. nov.

**Fig 5:**
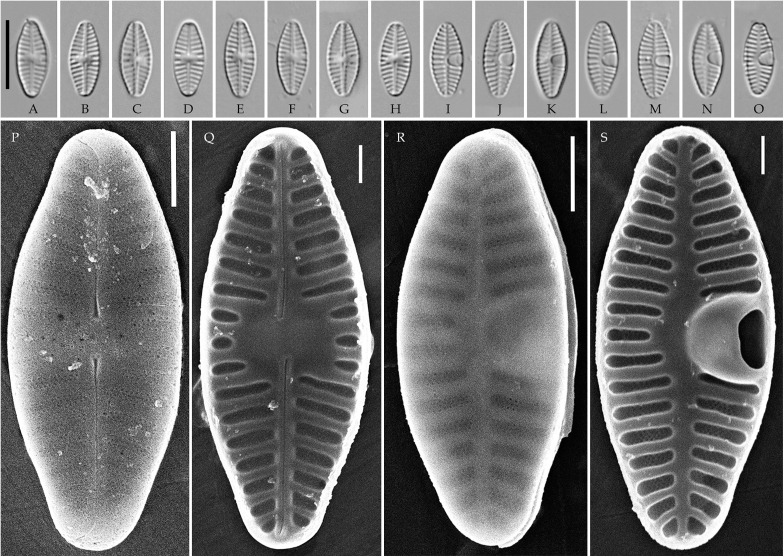
*Paraplanothidium laevis* sp. nov. Strain CBMCkam113, slide 07945, type material. (A–O) Light microscopy, differential interference contrast. (A–H) raphe valves; (I–O) rapheless valves. (B) holotype. Scale bar 10 μm. (P–S) Scanning electron microscopy. (P) raphe valve, external view; (Q) raphe valve, internal view; (R) rapheless valve, external view; (S) rapheless valve, internal view. Scale bar 2 μm (P, R), 1 μm (Q, S).

**Fig 6:**
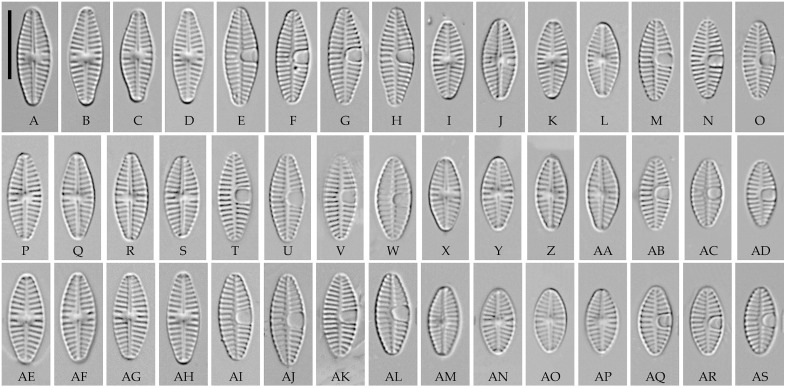
*Paraplanothidium laevis* sp. nov. (A–H) strain CBMCkam115, slide 07947; (I–O) strain CBMCkam128, slide 07960; (P–W) strain CBMCkam265, slide 08097; (X–AD) strain CBMCkam297, slide 08129; (AE–AL) strain CBMCkam322, slide 08154; (AM–AS) strain CBMCkam300, slide 08132. Light microscopy, differential interference contrast. (A–D, I–L, P–S, X–AA, AE–AH, AM–AP) raphe valves; (E–H, M–O, T–W, AB–AD, AI–AL, AQ–AS) rapheless valves. Scale bar 10 μm.

**Fig 7:**
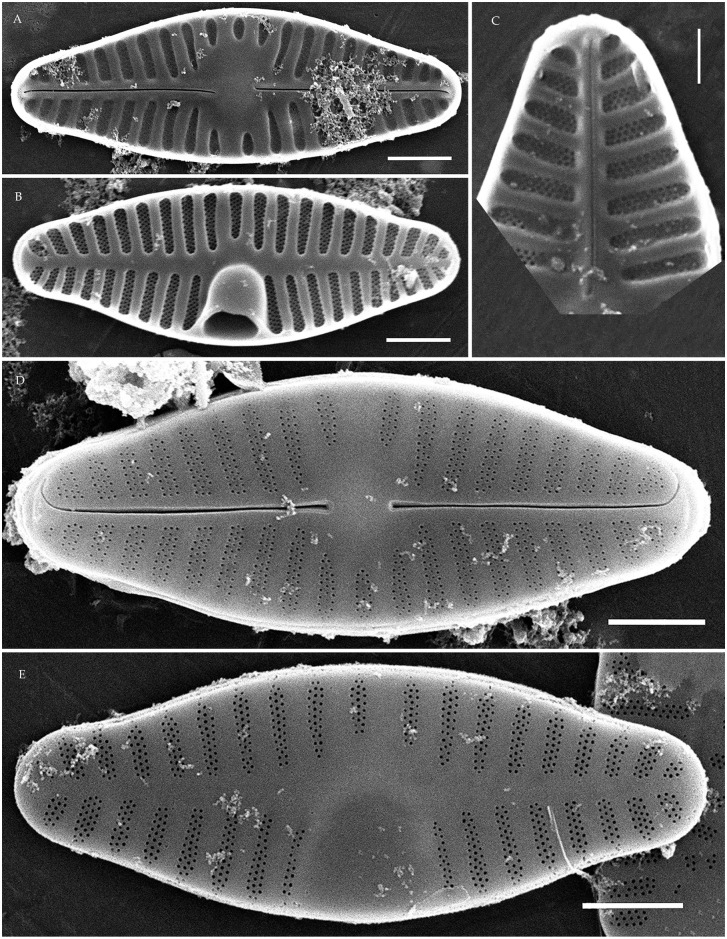
*Paraplanothidium laevis* sp. nov. (A, B, D, E) strain CBMCkam115; (C) strain CBMCkam128. Scanning electron microscopy. (A) raphe valve, internal view; (B) rapheless valve, internal view; (C) raphe valve, partial internal view of the valve end; (D) raphe valve, external view; (E) rapheless valve, external view. Scale bar 2 μm (A, B, D, E), 1 μm (C).

**Description. LM.** Frustules monoraphid. Valves linear-elliptic to lanceolate, with rounded or protracted ends. Raphe straight, filiform, central ends expanded. Axial area on the raphe valve usually narrow, central area variable. Axial area on the rapheless valve also narrow, a cavum (covered depression) is present on one side of the valve, in some species with a small semicircular central area on the other side.

**SEM.** External central raphe ends straight, expanded, distal ends turned to one side and extended onto the mantle. Internal central raphe ends slightly bent to opposite sides, distal ends terminate in small helictoglossae. Striae on both valves bi- to multiseriate, each consisting of up to 6 rows of small circular areolae. Internally, the interstriae are raised.

**Etymology**. The genus name refers to the similarity with *Planothidium* s. str.

#### *Paraplanothidium laevis* Tseplik, Glushchenko, Genkal, Maltsev et Kulikovskiy sp. nov. (Figs 5–7).

**Holotype**. Slide no. 07945 (represented here by Fig 5B), deposited in the Herbarium of K.A. Timiryazev Institute of Plant Physiology, Russian Academy of Sciences (HD), Moscow, Russia, prepared from oxidized culture strain CBMCkam113.

**Isotype**. Slide no. 07945b, Herbarium of Main Botanical Garden, Russian Academy of Science (MHA), Moscow, Russia.

**Reference strain**. CBMCkam113 from the Culture and Barcode Collection of Microalgae and Cyanobacteria “Algabank” (CBМC), isolated from sample 84.

**Type locality**. Russia, Lake Kurazhechnoye, sample 84, detritus and scrapes from stones (N 56.365.238 E 160.894.019), *leg*. M.S. Kulikovskiy & N.V. Lobus, 13.07.2021.

**Description. LM.** Valves lanceolate, with rounded to slightly protracted ends. Length 9.3–14.8 μm, width 4.3–5.6 μm. Raphe straight, filiform, central ends expanded. Axial area on the raphe valve narrow linear, central area absent or small and indistinct, roundish. Axial area on the rapheless valve narrow, linear to slightly lanceolate. A large cavum is present on one side of the rapheless valve. Striae on both valves parallel to weakly radiate in the centre, becoming radiate towards the ends, 13–17 in 10 μm.

**SEM**. External central raphe ends straight, expanded, distal ends turned to one side and extended onto the mantle. Internal central raphe ends slightly turned to opposite sides, distal ends terminate in faint helictoglossae. Striae multiseriate on both valves, consisting of 3–4 rows of small circular areolae. The external surface of the rapheless valve is unornamented. Internally, the interstriae are raised. A vestigial raphe is sometimes present on the inside of the rapheless valve. The cavum is large, more or less elongated, with a relatively small opening.

**Sequence data**. Partial 18S rDNA gene sequence comprising V4 domain sequences (GenBank accession number PX412385 for the strain CBMCkam113, PX412386 for the strain CBMCkam115, PX412387 for the strain CBMCkam128, PX412388 for the strain CBMCkam265, PX412389 for the strain CBMCkam297, PX412390 for the strain CBMCkam298, PX412391 for the strain CBMCkam300, PX412392 for the strain CBMCkam322) and partial *rbc*L sequences (GenBank accession number PX418650 for the strain CBMCkam113, PX418651 for the strain CBMCkam115, PX418652 for the strain CBMCkam128, PX418653 for the strain CBMCkam265, PX418654 for the strain CBMCkam297, PX418655 for the strain CBMCkam298, PX418656 for the strain CBMCkam300, PX418657 for the strain CBMCkam322).

**Etymology**. The epithet refers to the smooth, unornamented outer surface of the rapheless valves.

**Distribution**. As yet known only from the Kamchatka Peninsula, Russia.

### New combinations in the genus Paraplanothidium gen. nov.

#### *Paraplanothidium abbreviatum* (Reimer) Tseplik, Glushchenko, Genkal, Maltsev et Kulikovskiy comb. et stat. nov.

**Basionym**: *Achnanthes lanceolata* var*. abbreviata* Reimer 1966. Consideration of fifteen diatom taxa (Bacillariophyta) from the Savannah River, including seven described as new. Notulae Naturae (Academy of Natural Sciences of Philadelphia) 397: 3; pl. 1, Figs 6–9.

**Type**:—USA, South Carolina, Allendale Co., Savannah R. (holotype: ANSP GC43857a, see Potapova, 2012: 41, figs 109–110).

**Synonym**: *Pl*anothidium abbreviatum** (Reimer) Potapova 2012, Diatom Research 27(1–2): 40, figs 104–110.

#### *Paraplanothidium africanum* (Van de Vijver et al.) Tseplik, Glushchenko, Genkal, Maltsev et Kulikovskiy comb. nov.

**Basionym**: *Planothidium africanum* Van de Vijver et al. 2023. *Planothidium africanum* sp. nov., a new freshwater diatom (Bacillariophyta) species from Tanzania. Phytotaxa 585(4): 282, figs 1–35.

**Type**:—Africa, Tanzania, Morogoro (holotype: BR-4778 = Fig 6).

#### *Paraplanothidium alekseevae* (Gogorev et Lange) Tseplik, Glushchenko, Genkal, Maltsev et Kulikovskiy comb. nov.

**Basionym**: *Planothidium alekseevae* Gogorev et Lange 2015. Achnanthoid diatoms (Bacillariophyta) of the relict Lake Mogilnoye (Kildin Island, Barents Sea). Nov. Sist. Nizsh. Rast. 49: 15, pl. I(1–15), II(34–5).

**Type**:—Russia, Barents Sea, Kildin Island, Lake Mogilnoye (holotype: G18—LE, Plate I, *9, 13*).

#### *Paraplanothidium apiculatum* (R.M. Patrick) Tseplik, Glushchenko, Genkal, Maltsev et Kulikovskiy comb. et stat. nov.

**Basionym**: *Achnanthes lanceolata* var. *apiculata* R.M. Patrick 1945. A taxonomic and ecological study of some diatoms from the Pocono Plateau and adjacent regions. Farlowia 2(2): 167, pl. 1(4–5).

**Type**:—USA, Pennsylvania, Shohola Falls, Pike County (holotype: J. Burke A-2190, Pl. I, figs 4, 5).

**Synonyms**: *Planothidium apiculatum* (R.M. Patrick) Lange-Bertalot 1999, Iconographia Diatomologica 6: 280.

#### *Paraplanothidium aueri* (Krasske) Tseplik, Glushchenko, Genkal, Maltsev et Kulikovskiy comb. nov.

**Basionym**: *A*chnanthes aueri** Krasske 1949. Subfossile Diatomeen aus den Mooren Patagoniens und Feuerlands. Suomalaisen Tiedeakatemian Toimituksia. Annales Academiae Scientiarum Fennicae, Sarja Series A. IV, Biologica. 14: 77, fig. 3.

**Type**:—Chile, Tierra del Fuego (lectotype: D III 879, see Lange-Bertalot et al. 1996: 24).

**Synonyms**: *Planothidium aueri* (Krasske) Lange-Bertalot 1999, Iconographia Diatomologica 6: 275; *Achnantheiopsis aueri* (Krasske) Lange-Bertalot 1997, Arch. Protistenk. 148: 206.

#### *Paraplanothidium bagualense* (C.E. Wetzel et Ector) Tseplik, Glushchenko, Genkal, Maltsev et Kulikovskiy comb. nov.

**Basionym**: *Planothidium bagualense* C.E. Wetzel et Ector 2014. Taxonomy, distribution and autecology of *Planothidium bagualensis* sp. nov. (Bacillariophyta) a common monoraphid species from southern Brazil rivers. Phytotaxa 156(4): 203, figs 2–19.

**Type**:—Brazil, Rio Grande do Sul, Sinimbú, Pardinho Falls (‘Salto do Pardinho’), Pardinho River, Rio Pardo hydrographical basin (holotype: BR-4316 = Fig 4).

#### *Paraplanothidium baicalrostratum* (Kulikovskiy et Lange-Bertalot) Tseplik, Glushchenko, Genkal, Maltsev et Kulikovskiy comb. nov.

**Basionym**: *Pla*nothidium baicalrostratum** Kulikovskiy et Lange-Bertalot in Kulikovskiy et al. 2015. Lake Baikal: hotspot of endemic diatoms II. Iconographia Diatomologica 26: 60, pl. 71: figs 1–45.

**Type**:—Russia, Lake Baikal, Bolshoi Ushkaniy Island (holotype: IBIW 15645m = Fig. 71: 1).

#### *Paraplanothidium biporomum* (M.H. Hohn et Hellerman) Tseplik, Glushchenko, Genkal, Maltsev et Kulikovskiy comb. nov.

**Basionym**: *Achnanthes biporoma* M.H. Hohn et Hellerman 1963. The taxonomy and structure of diatom populations from three eastern North American rivers using three sampling methods. Transactions of the American Microscopical Society 82 (3): 273, pl. 2: figs 5, 6.

**Type**:—USA, Savannah River, Burke Co.-Barnwell Co., Georgia-North Carolina (holotype: G.C. 442261a, Pl. 11, Fig. 5, 6).

**Synonyms**: *Achnanthes lanceolata* subsp. *biporoma* (M.H. Hohn et Hellerman) Lange-Bertalot 1993, Bibliotheca Diatomologica 27: 3; *Achnanthidium biporomum* (M.H. Hohn et Hellerman) Czarnecki in Czarnecki, Edlund 1995, Diatom Research 10(1): 208; *Achnantheiopsis biporoma* (M.H. Hohn et Hellerman) Lange-Bertalot 1997, Arch. Protistenk. 148: 206; *Planothidium biporomum* (M.H. Hohn et Hellerman) Lange-Bertalot 1999, Iconographia Diatomologica 6: 275.

#### *Paraplanothidium borneolanceolatum* (Lange-Bertalot) Tseplik, Glushchenko, Genkal, Maltsev et Kulikovskiy comb. nov.

**Basionym**: *Achnantheiopsis borneolanceolata* Lange-Bertalot 1997. Zur Revision der Gattung *Achnanthes* sensu lato (Bacillariophyceae): *Achnantheiopsis*, eine neue Gattung mit dem Typus generis *A. lanceolata*. Arch. Protistenk. 148: 208.

**Type**:—Borneo, Xipungit Falls, Poring (holotype: Praep. no. As-68, see Lange-Bertalot and Krammer 1989, Fig. 85: 11–14, Fig. 91: 2).

**Synonyms**: *Planothidium borneolanceolatum* (Lange-Bertalot) Lange-Bertalot 1999, Iconographia Diatomologica 6: 275.

#### *Paraplanothidium boudoui* (Metzeltin et Lange-Bertalot) Tseplik, Glushchenko, Genkal, Maltsev et Kulikovskiy comb. nov.

**Basionym**: *Achnanthes boudoui* Metzeltin et Lange-Bertalot 1998. Tropical diatoms of South America I: About 700 predominantly rarely known or new taxa representative of the neotropical flora. Iconographia Diatomologica 5: 18, pl. 69: figs 9–14; pl. 70: figs 1, 4.

**Type**:—Brazil, Rio Tapajós near Santarém (holotype: Praep. AmS-573, Fig. 69: 9–14, 70: 1, 4).

**Synonyms**: *Planothidium boudoui* (Metzeltin et Lange-Bertalot) M. Coste, Eulin-Garrigue, O. Monnier et Delmas in Coste et al. 2024, Notulae Algarum 32: 1, figs 1–8.

#### *Paraplanothidium brasiliense (*C.E. Wetzel et S. Blanco) Tseplik, Glushchenko, Genkal, Maltsev et Kulikovskiy comb. nov.

**Basionym**: *Planothidium brasiliense* C.E. Wetzel et S. Blanco in Wetzel et al. 2019. On some common and new cavum-bearing *Planothidium* (Bacillariophyta) species from freshwater. Fottea 19(1): 58, figs 211–217.

**Type**:—Brazil, Ribeirão das Ostras, Eldorado, São Paulo (holotype: SP–255765, Figs 211–217).

#### *Paraplanothidium cavilanceolatum* (Wetzel, M.G. Kelly et Van de Vijver) Tseplik, Glushchenko, Genkal, Maltsev et Kulikovskiy comb. nov.

**Basionym**: *P*lanothidium cavilanceolatum** Wetzel, M.G. Kelly et Van de Vijver in Wetzel et al. 2019. On some common and new cavum-bearing *Planothidium* (Bacillariophyta) species from freshwater. Fottea 19(1): 62, figs 464–521.

**Type**:—Belgium, Flanders, Kleine Beek – Heidebeek (holotype: BR–4533, figs 464–521).

#### *Paraplanothidium chilense* (Hustedt) Tseplik, Glushchenko, Genkal, Maltsev et Kulikovskiy comb. nov.

**Basionym**: *Achn*anthes chilensis** Hustedt 1927. Fossile Bacillariaceen aus dem Loa-Becken in der Atacama-Wüste, Chile. Archiv für Hydrobiologie 18(2): 238, pl. 7, figs. 3–4.

**Type**:—Chile, Atacama Desert (holotype: Ma1/5, see Simonsen 1987, Pl. 163: 5–11).

**Synonyms**: *Achnantheiopsis chilensis* (Hustedt) Lange-Bertalot 1997, Arch. Protistenk. 148: 206; *Planothidium chilense* (Hustedt) Lange-Bertalot 1999, Iconographia Diatomologica 6: 27.

#### *Paraplanothidium comperei* (C.E. Wetzel, N’Guessan et Tison-Rosebery) Tseplik, Glushchenko, Genkal, Maltsev et Kulikovskiy comb. nov.

**Basionym**: *Planothidium comperei* C.E. Wetzel, N’Guessan et Tison-Rosebery in N’Guessan et al. 2014. *Planothidium comperei* sp. nov. (Bacillariophyta), a new diatom species from Ivory Coast. Plant Ecology and Evolution 147(3): 456–457, figs 1A–M, 2.

**Type**:—Africa, Ivory Coast, Mborou, River M’Borou, River Agneby hydrographical basin (holotype: BR-4376 = Fig 1B).

#### *Paraplanothidium curtistriatum* (C.E. Wetzel, Van de Vijver et Ector) Tseplik, Glushchenko, Genkal, Maltsev et Kulikovskiy comb. nov.

**Basionym**: *Planothidium curtistriatum* C.E. Wetzel, Van de Vijver et Ector in Wetzel et al. 2019. On some common and new cavum-bearing *Planothidium* (Bacillariophyta) species from freshwater. Fottea 19(1): 61, figs 386–414, 444–456.

**Type**:—France, River Beaume at Le Brignon, Auvergne–Rhône–Alpes region (holotype: BR–4532, figs 386–414, 444–456).

#### *Paraplanothidium dyakonovii* (Tseplik, Glushchenko, Maltsev et Kulikovskiy) Tseplik, Glushchenko, Genkal, Maltsev et Kulikovskiy comb. nov.

**Basionym**: *Planothidium dyakonovii* Tseplik, Glushchenko, Maltsev et Kulikovskiy in Tseplik et al. 2024. New cavum-bearing *Planothidium* (Achnanthidiaceae, Bacillariophyceae) species from the Kamchatka Peninsula, Russia. Phytotaxa 665 (3): 277, figs 23–41.

**Type**:—Russia, Kamchatka Peninsula, Dachnye hot springs (holotype: 08205 = Fig. 24).

#### *Paraplanothidium frequentissimum* (Lange-Bertalot) Tseplik, Glushchenko, Genkal, Maltsev et Kulikovskiy comb. et stat. nov.

**Basionym**: *Achnanthes lanceolata* subsp. *frequentissima* Lange-Bertalot 1993. 85 Neue Taxa und über 100 weitere neu definierte Taxa ergänzend zur Süßwasserflora von Mitteleuropa Vol. 2/1–4. Bibliotheca Diatomologica, 27: 4.

**Type**:—Belgium, Brussels (holotype: n° 236, see Wetzel et al. 2019, Figs 251–282, 344–353).

**Synonyms**: *A*chnanthes lanceolata** var. *dubia* f. *minuta* Grunow in Van Heurck 1882: 65; *Achnantheiopsis frequentissima* (Lange-Bertalot) Lange-Bertalot 1997, Arch. Protistenk. 148: 201; *Planothidium frequentissimum* (Lange-Bertalot) Lange-Bertalot 1999, Iconographia Diatomologica 6: 282.

#### *Paraplanothidium gallicum* (C.E. Wetzel et Ector) Tseplik, Glushchenko, Genkal, Maltsev et Kulikovskiy comb. nov.

**Basionym**: *Planothidium gallicum* C.E. Wetzel et Ector in Wetzel et al. 2019. On some common and new cavum-bearing *Planothidium* (Bacillariophyta) species from freshwater. Fottea 19 (1): 56, figs 164–185, 224–229.

**Type**:—France, Saunus River at Magescq, Landes department in Nouvelle-Aquitaine, south-western France (holotype: BR–4528, figs 164–185, 224–229).

#### *Paraplanothidium hinzianum* (C.E. Wetzel, Van de Vijver et Ector) Tseplik, Glushchenko, Genkal, Maltsev et Kulikovskiy comb. nov.

**Basionym**: *Planothidium hinzianum* C.E. Wetzel, Van de Vijver et Ector in Wetzel et al. 2019. On some common and new cavum-bearing *Planothidium* (Bacillariophyta) species from freshwater. Fottea 19 (1): 61, figs 415–439, 457–463.

**Type**:—Germany, Wümme River, Borgfeld (Bremen), epiphyte on Hypnum (holotype: 212/73 BRM, figs 415–439, 457–463).

#### *Paraplanothidium incuriatum* (C.E. Wetzel, Van de Vijver et Ector) Tseplik, Glushchenko, Genkal, Maltsev et Kulikovskiy comb. nov.

**Basionym**: *Planothidium incuriatum* C.E.Wetzel, Van de Vijver et Ector in Wetzel et al. 2013. *Planothidium incuriatum* sp. nov. a widely distributed diatom species (Bacillariophyta) and type analysis of *Planothidium biporomum*. Phytotaxa 138 (1): 49, figs 19–36, 51–89.

**Type**:—France, Île de France Region (Yvelines), Magny-les-Hameaux: ‘Croix au Buis’ creek, Yvette River basin (holotype: BR-4315 = Fig. 21)

#### *Paraplanothidium infrequens* (Lange-Bertalot et U. Rumrich) Tseplik, Glushchenko, Genkal, Maltsev et Kulikovskiy comb. nov.

**Fig 8:**
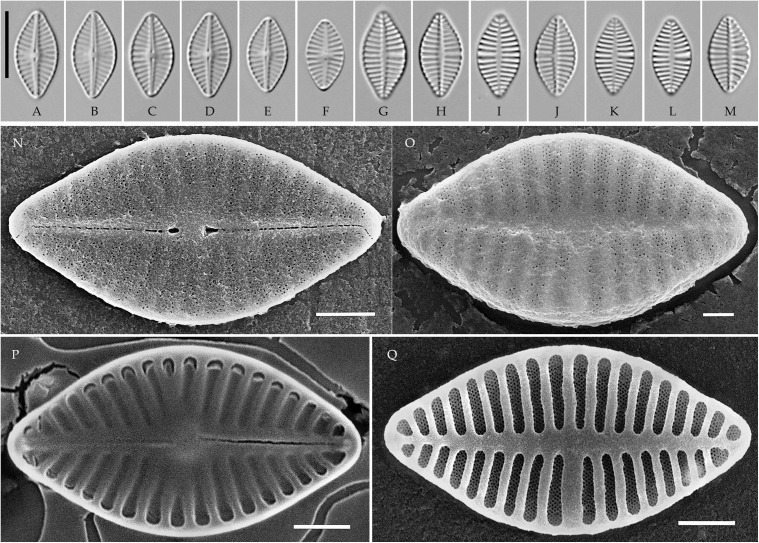
*Pseudoplanothidium foliiformis* sp. nov. Strain CBMCkam470, slide 08301, type material. (A–M) Light microscopy, differential interference contrast. (A–F) raphe valves; (G–M) rapheless valves. (A) holotype. Scale bar 10 μm. (N–Q) Scanning electron microscopy. (N) raphe valve, external view; (O) rapheless valve, external view; (P) raphe valve, internal view; (Q) rapheless valve, internal view. Scale bar 2 μm (N, P, Q), 1 μm (O).

**Fig 9:**
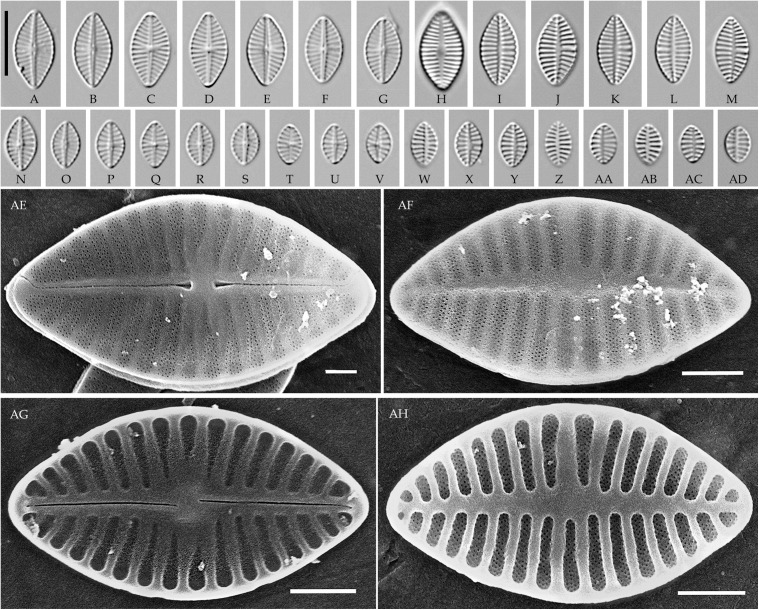
*Pseudoplanothidium foliiformis* sp. nov. (A–M, AE–AH) strain CBMCkam430, slide 08262; (N–AD) strain CBMCkam483, slide 08314. (A–AD) Light microscopy, differential interference contrast. (A–G, N–V) raphe valves; (H–M, W–AD) rapheless valves. Scale bar 10 μm. (AE–AH) Scanning electron microscopy. (AE) raphe valve, external view; (AF) rapheless valve, external view; (AG) raphe valve, internal view; (AH) rapheless valve, internal view. Scale bar 2 μm (AF–AH), 1 μm (AE).

**Basionym:** *Planothid*ium infrequens** Lange-Bertalot et U. Rumrich in Rumrich et al. 2000. Diatomeen der Anden von Venezuela bis Patagonien/Feuerland und zwei weitere Beiträge. Diatoms of the Andes from Venezuela to Patagonia/Tierra del Fuego and two additional contributions. Iconographia Diatomologica 9: 212, pl. 27: figs 3–9.

**Type**:—Chile, Altiplano, Río Lauca (holotype: AmS-Ch 60, pl. 27: figs 3–9).

#### *Paraplanothidium lagerheimii* (P.T. Cleve) Tseplik, Glushchenko, Genkal, Maltsev et Kulikovskiy comb. nov.

**Basionym**: *C*occoneis lagerheimii** P.T. Cleve 1894. Les Diatomées de l’Equateur. Le Diatomiste 2 (18): 100.

**Type**:—Ecuador, San-Nicolas, Los Ríos (see Cleve 1894).

**Synonyms**: *Planothidium lagerheimii* (P.T. Cleve) Wetzel et Ector 2014, Phytotaxa 188 (5): 263, figs 1–20.

#### *Paraplanothidium magnificum* (Hustedt) Tseplik, Glushchenko, Genkal, Maltsev et Kulikovskiy comb. nov.

**Basionym:** *A*chnanthes magnifica** Hustedt 1952. Neue und wenig bekannte Diatomeen. IV. Botaniska Notiser 4: 385, figs 62–65.

**Type**:—Brazil, Itatiaia Mountains (holotype: BRM 323/68, see Simonsen 1987, p. 384).

**Synonyms**: *P*lanothidium magnificum** (Hustedt) Lange-Bertalot 1999, Iconographia Diatomologica 6: 277.

#### *Paraplanothidium makarevichae* (Kulikovskiy et Lange-Bertalot) Tseplik, Glushchenko, Genkal, Maltsev et Kulikovskiy comb. nov.

**Basionym**: *Planot*hidium makarevichae** Kulikovskiy et Lange-Bertalot in Kulikovskiy et al. 2015. Lake Baikal: hotspot of endemic diatoms II. Iconographia Diatomologica 26: 63, pl. 72, figs 1–14.

**Type**:—Russia, Lake Baikal, Bolshoi Ushkaniy Island (holotype: IBIW 15645m = Fig. 72: 1).

#### *Paraplanothidium marganaiense* (Lai, Ector et C. Wetzel) Tseplik, Glushchenko, Genkal, Maltsev et Kulikovskiy comb. nov.

**Basionym**: *Planothidium marganaiense* Lai, Ector et C. Wetzel in Lai et al. 2021. *Planothidium margalaiensis* sp. nov. (Bacillariophyta), a new cavum-bearing species from a karst spring in south-western Sardinia (Italy). Phytotaxa 489 (2): 143, figs 2–57.

**Type**:—Italy, Carbonia-Iglesias, San Giovanni cave at Domusnovas (Monte Acqua, Marganai massif) (holotype: BR-4636 = Fig. 9).

#### *Paraplanothidium minuanum* (M.G. Junqueira, L.O. Crossetti et C.E. Wetzel) Tseplik, Glushchenko, Genkal, Maltsev et Kulikovskiy comb. nov.

**Basionym**: *P*lanothidium minuanum** M.G. Junqueira, L.O. Crossetti et C.E. Wetzel 2024. *Planothidium minuanum* sp. nov., a new diatom (Bacillariophyta) species from southern Brazilian coastal lakes. Phytotaxa 676 (2): 129, figs 2–56.

**Type**:—Brazil, Rio Grande do Sul, Balneário Pinhal, Cerquinha coastal lake (holotype: ICN-212270 = Fig. 6).

#### *Paraplanothidium miotum* (Carter et Denny) Tseplik, Glushchenko, Genkal, Maltsev et Kulikovskiy comb. nov.

**Basionym**: *A*chnanthes miota** Carter et Denny 1982. Freshwater algae of Sierra Leone III. Bacillariophyceae: Part (i) Diatoms from the River Jong (Taia) at Njala. In: Diatomaceae III. Nova Hedwigia, Beihefte 73: 286, pl. 1, fig. 9.

**Type**:—Africa, Sierra Leone, River Jong at Njala (holotype: BM78107, see N’Guessan et al. 2014, Fig. 1N–Y).

**Synonyms**: *Achnantheiopsis miota* (Carter et Denny) Lange-Bertalot 1997, Arch. Protistenk. 148: 207; *Planothidium miotum* (Carter et Denny) Lange-Bertalot 1999, Iconographia Diatomologica 6: 278.

#### *Paraplanothidium naradoense* (Jahn et Zimmermann) Tseplik, Glushchenko, Genkal, Maltsev et Kulikovskiy comb. nov.

**Basionym**: *Pla*nothidium naradoense** Jahn et Zimmermann in Jahn et al. 2017. *Planothidium lanceolatum* and *Planothidium frequentissimum* reinvestigated with molecular methods and morphology: four new species and the taxonomic importance of the sinus and cavum. Diatom Research 32 (1): 100–101.

**Type**:—Korea, ChollaNamdo, NaeNarado Island (holotype: B 40 0040800, strain D23_024 = Fig. 352).

#### *Paraplanothidium piaficum* (Carter et Denny) Tseplik, Glushchenko, Genkal, Maltsev et Kulikovskiy comb. nov.

**Fig 10:**
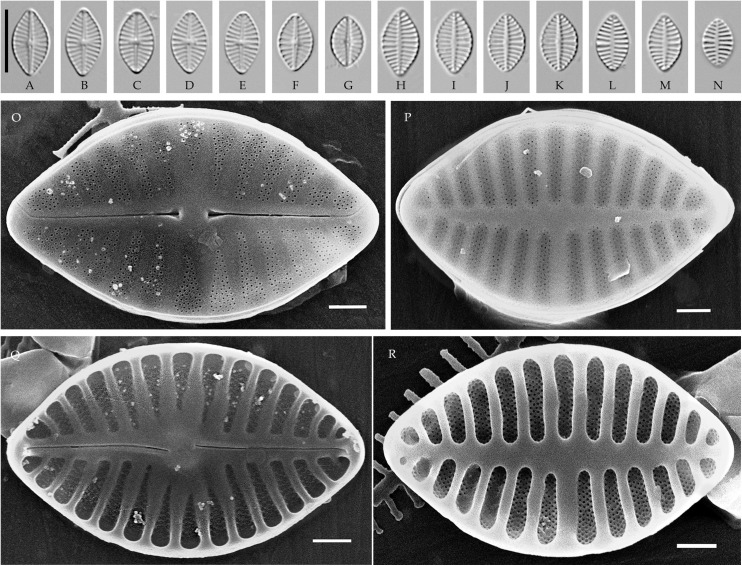
*Pseudoplanothidium foliiformis* sp. nov. Strain CBMCkam446, slide 08278. (A–N) Light microscopy, differential interference contrast. (A–G) raphe valves; (H–N) rapheless valves. Scale bar 10 μm. (O–R) Scanning electron microscopy. (O) raphe valve, external view; (P) rapheless valve, external view; (Q) raphe valve, internal view; (R) rapheless valve, internal view. Scale bar 1 μm.

**Basionym**: *A*chnanthes piafica** Carter et Denny 1982. Freshwater algae of Sierra Leone III. Bacillariophyceae: Part (i) Diatoms from the River Jong (Taia) at Njala. In: Diatomaceae III. Nova Hedwigia, Beihefte 73: 286, pl. 1: fig. 10.

**Type**:—Africa, Sierra Leone, River Jong at Njala (holotype: BM78108, see N’Guessan et al. 2014, Fig. 3A–G, I–L).

**Synonyms**: *Pla*nothidium piaficum** (Carter et Denny) C.E. Wetzel et Ector in N’Guessan et al. 2014, Plant Ecology and Evolution 147 (3): 456, figs 3 A–G, I–L.

#### *Paraplanothidium potapovae* (C.E. Wetzel et Ector) Tseplik, Glushchenko, Genkal, Maltsev et Kulikovskiy comb. nov.

**Basionym**: *Pl*anothidium potapovae** C.E. Wetzel et Ector in Wetzel et al. 2019. On some common and new cavum-bearing *Planothidium* (Bacillariophyta) species from freshwater. Fottea 19 (1): 57, figs 200–210, 239–246–246.

**Type**:—France, River La Boute Vive at Sainte-Montaine, Centre-Val de Loire region (holotype: BR-4529, Figs 200–210, 239–246–246).

#### *Paraplanothidium pseudotanense* (A. Cleve) Tseplik, Glushchenko, Genkal, Maltsev et Kulikovskiy comb. nov.

**Basionym***: Achn*anthes pseudotanensis** A. Cleve in Cleve-Euler 1953. Die Diatomeen von Schweden und Finnland. Teil III. Monoraphideae, Biraphideae 1. Kungliga Svenska Vetenskapsakademiens Handlingar, ser. IV 4 (5): 25, fig. 524.

**Type**:—Sweden, Örträsk (see Cleve-Euler 1953: 25).

**Synonyms:** *P*lanothidium pseudotanense** (A. Cleve) Lange-Bertalot 1999, Iconographia Diatomologica 6: 278.

#### *Paraplanothidium rostratoholarcticum* (Lange-Bertalot et Bąk) Tseplik, Glushchenko, Genkal, Maltsev et Kulikovskiy comb. nov.

**Basionym**: *P*lanothidium rostratoholarcticum** Lange-Bertalot et Bąk 2015. Four small-celled *Planothidium* species from Central Europe proposed as new to science. Oceanological and Hydrobiological Studies 43 (4): 354, figs 8a–s, 9a–g.

**Type**:—Germany, Bremen (lectotype: BRM 212/73, see Simonsen 1987, Lange-Bertalot & Bąk 2015: 356).

#### *Paraplanothidium rostratum* (Østrup) Tseplik, Glushchenko, Genkal, Maltsev et Kulikovskiy comb. nov.

**Basionym**: *A*chnanthes rostrata** Østrup 1902. Freshwater diatoms. In: Flora of Koh Chang. Contributions to the knowledge of the vegetation in the Gulf of Siam by Johs. Schmidt. Part VII. Botanisk Tidsskrift 25 (1): 253, pl. 1, fig. 11.

**Type**:—Thailand, Koh Chang Island, Klong Sarlakpet (holotype: 3293 (C), see Wetzel et al. 2019: 52, Figs 11–22).

**Synonyms**: *M*icroneis lanceolata** var. *rostrata* (Østrup) Schulz 1928, Bericht der Westpreussischen Botanisch-Zoologischen Verein 50: 138; *Achnanthes lanceolata* f. *rostrata* (Østrup) Hustedt 1957, Abhandlungen der Naturwissenschaftlichen Verein zu Bremen 34 (3): 251; *Achnanthes lanceolata* subsp. *rostrata* (Østrup) Lange-Bertalot in Krammer, Lange-Bertalot 1991: 79; *Achnantheiopsis rostrata* (Østrup) Lange-Bertalot 1997, Arch. Protistenk. 148: 208, fig. 23; *Planothidium rostratum* (Østrup) Lange-Bertalot 1999, Iconographia Diatomologica 6: 279.

#### *Paraplanothidium scrobiculatum* (Marquardt et C.E. Wetzel) Tseplik, Glushchenko, Genkal, Maltsev et Kulikovskiy comb. nov.

**Basionym**: *Planothidium scrobiculatum* Marquardt et C.E. Wetzel in Marquardt et al. 2021. *Planothidium scrobiculatum* sp. nov. (Bacillariophyta), a new monoraphid diatom from freshwater Pleistocene deposits of South America. Fottea 21 (1): 55, figs 1–48.

**Type**:—Brazil, São Paulo, Parelheiros District: Colônia basin, sediment core (holotype: SP365549 = Fig. 5, 21).

#### *Paraplanothidium sheathii* (Stancheva) Tseplik, Glushchenko, Genkal, Maltsev et Kulikovskiy comb. nov.

**Basionym**: *P*lanothidium sheathii** Stancheva 2019. *Planothidium sheathii*, a new monoraphid diatom species from rivers in California, USA. Phytotaxa 393 (2): 132, figs 1–29.

**Type**:—USA, California, San Joaquin River (holotype: GC 65325 = Fig. 7).

#### *Paraplanothidium straubianum* (C.E. Wetzel, Van de Vijver et Ector) Tseplik, Glushchenko, Genkal, Maltsev et Kulikovskiy comb. nov.

**Basionym**: *Pl*anothidium straubianum** C.E.Wetzel, Van de Vijver et Ector in Wetzel et al. 2019. On some common and new cavum-bearing *Planothidium* (Bacillariophyta) species from freshwater. Fottea 19 (1): 60, figs 362–385, 440–443.

**Type**:—Switzerland, Lac des Taillères, Neuchâtel (holotype: BR-4531, Figs 362–385, 440–443).

#### *Paraplanothidium tujii* (C.E. Wetzel et Ector) Tseplik, Glushchenko, Genkal, Maltsev et Kulikovskiy comb. nov.

**Basionym**: *Planothidium tujii* C.E. Wetzel et Ector in Wetzel et al. 2019. On some common and new cavum-bearing *Planothidium* (Bacillariophyta) species from freshwater. Fottea 19 (1): 56, figs 140–163, 218–223.

**Type**:—Japan, Yamagata Prefecture, Yuza, River Araizawa, material TNS–AL–61807, strain Ak807 (holotype: prep. n° 114, see Tuji 2014: 15).

#### *Paraplanothidium tyushovii* (Tseplik, Glushchenko, Maltsev et Kulikovskiy) Tseplik, Glushchenko, Genkal, Maltsev et Kulikovskiy comb. nov.

**Basionym**: *P*lanothidium tyushovii** Tseplik, Glushchenko, Maltsev et Kulikovskiy in Tseplik et al. 2024. New cavum-bearing *Planothidium* (Achnanthidiaceae, Bacillariophyceae) species from the Kamchatka Peninsula, Russia. Phytotaxa 665 (3): 276, figs 4–22.

**Type**:—Russia, Kamchatka Peninsula, Greshnaya river (holotype: 08120 = Fig. 4).

#### *Paraplanothidium victorii* (P.M. Novis, J. Braidwood et C. Kilroy) Tseplik, Glushchenko, Genkal, Maltsev et Kulikovskiy comb. nov.

**Basionym**: *Planothidium victorii* P.M. Novis, J. Braidwood et C. Kilroy 2012. Small diatoms (Bacillariophyta) in cultures from the Styx River, New Zealand, including descriptions of three new species. Phytotaxa 64: 22, figs 26–41, 161.

**Type**:—New Zealand, Canterbury, Styx River (holotype: CHR618408, strain LCR-S:18:1:1, Figs 26–41, 161; epitype: B 40 0040871, strain D06_014, see Jahn et al. 2017, figs 240–250).

#### *Paraplanothidium wetzelii* (Schimani, N.Abarca et R. Jahn) Tseplik, Glushchenko, Genkal, Maltsev et Kulikovskiy comb. nov.

**Basionym**: *Planothidium wetzelii* Schimani, N.Abarca et R. Jahn in Juchem et al. 2023. Lipid degradation and photosynthetic traits after prolonged darkness in four Antarctic benthic diatoms, including the newly described species *Planothidium wetzelii* sp. nov. Frontiers in Microbiology 14:1241826, figs 3, 4.

**Type**:—South Shetland Islands, King George Island, Potter Cove (holotype: B 40 0045341a, strain D300_025 = Figs 3BJ, 3BK).

#### *Paraplanothidium xinguense* (K.S. Morais, C.E. Wetzel et C.E.M. Bicudo) Tseplik, Glushchenko, Genkal, Maltsev et Kulikovskiy comb. nov.

**Basionym**: *Planothidium xinguense* K.S. Morais, C.E. Wetzel et C.E.M. Bicudo in Morais et al. 2020. A new *Planothidium* species (Achnanthidiaceae, Bacillariophyceae) from Xingú Ria, Amazon River basin, Brazil. Phytotaxa 477 (2): 197, figs 2–29.

**Type**:—Brazil, Pará State, Porto de Moz, Xingu River (holotype: SP 513829, figs 2–29).

### *Pseudoplanothidium* Tseplik, Glushchenko, Genkal, Maltsev et Kulikovskiy gen. nov. (Figs 8–11)

**Fig 11:**
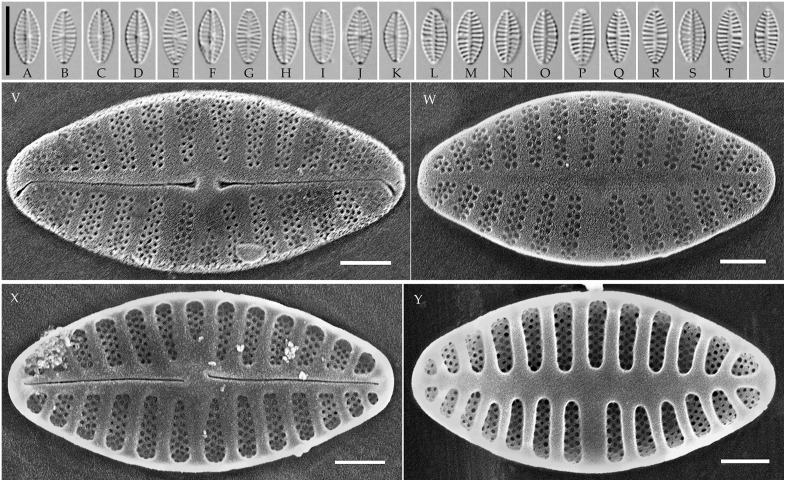
*Pseudoplanothidium minutum* sp. nov. Strain CBMCkam478, slide 08309, type material. (A–U) Light microscopy, differential interference contrast. (A–K) raphe valves; (L–U) rapheless valves. (C) holotype. Scale bar 10 μm. (V–Y) Scanning electron microscopy. (V) raphe valve, external view; (W) rapheless valve, external view; (X) raphe valve, internal view; (Y) rapheless valve, internal view. Scale bar 1 μm.

**Type species (here designated)**. *Ps*eudoplanothidium foliiformis** sp. nov.

**Description. LM.** Frustules monoraphid. Valves lanceolate to elliptic-lanceolate, with rounded or slightly protracted ends. Raphe straight, filiform, central ends expanded. Axial area on the raphe valve generally narrow, central area variable. Axial area on the rapheless valve also narrow, central area absent or represented by the central striae spaced wider than the rest on one side of the valve.

**SEM.** External central raphe ends straight, expanded, distal ends turned to one side and extended onto the mantle. Internal central raphe ends slightly bent to opposite sides, distal ends terminate in small helictoglossae. Striae on both valves bi- to multiseriate, consisting of up to 6 rows of small circular areolae per stria. In some species with triseriate striae, the areolae in the central row are smaller than in the other two rows; otherwise, all areolae are of similar size. Internally, the interstriae are raised (Fig 8**–**11).

**Etymology**. The genus name refers to the similarity with *Planothidium* s. str.

#### *Pseudoplanothidium foliiformis* Tseplik, Glushchenko, Genkal, Maltsev et Kulikovskiy sp. nov. (Figs 8–10).

**Holotype**. Slide no. 08301 (represented here by Fig 8A), deposited in the Herbarium of K.A. Timiryazev Institute of Plant Physiology, Russian Academy of Sciences (HD), Moscow, Russia, prepared from oxidized culture strain CBMCkam470.

**Isotype**. Slide no. 08301b, Herbarium of Main Botanical Garden, Russian Academy of Science (MHA), Moscow, Russia.

**Reference strain**. CBMCkam470 from the Culture and Barcode Collection of Microalgae and Cyanobacteria “Algabank” (CBМC), isolated from sample 167.

**Type locality.** Russia, Yuzhnaya Bay, sample 167, benthos and detritus (N 52.952.434 E 158.687.17), *leg*. M.S. Kulikovskiy & N.V. Lobus, 17.07.2021.

**Description**. **LM**. Valves widely lanceolate, with rounded or very slightly protracted ends. Length 5.7–13.5 μm, width 3.9–6.9 μm. Raphe filiform, straight, with expanded central ends. Axial area on the raphe valve narrow linear, central area almost absent or small and rounded. Axial area on the rapheless valve also narrow linear, central area mostly absent, two central striae may be spaced wider than the rest on one side of the valve. Striae on both valves parallel or almost parallel at the centre of the valve, becoming strongly radiate towards the ends, 13–18 in 10 μm on the raphe valve, 14–17 in 10 μm on the rapheless valve.

**SEM**. External central raphe ends straight, expanded, distal ends turned to one side and extended onto the mantle. Internal central raphe ends slightly bent to opposite sides, distal ends terminate in small helictoglossae. Striae multiseriate on both valves, consisting of 4–5 rows of areolae per stria on the rapheless valve and on the raphe valve there are 4–5 rows of areolae per stria near the valve margin and 1–3 rows per stria new the axial area. The areolae are small, round. The rapheless valve does not have any distinct patterns of ornamentation; the axial area may be slightly depressed or not. Internally the interstriae are distinctly raised.

**Sequence data**. Partial 18S rDNA gene sequence comprising V4 domain sequences (GenBank accession number PX412393 for the strain CBMCkam430, PX412394 for the strain CBMCkam446, PX412395 for the strain CBMCkam470, PX412397 for the strain CBMCkam483) and partial *rbc*L sequences (GenBank accession number PX418658 for the strain CBMCkam430, PX418659 for the strain CBMCkam446, PX418660 for the strain CBMCkam470, PX418662 for the strain CBMCkam483).

**Etymology**. The epithet *foliiformis* (Lat. leaf-like) refers to the valve outline of the new species resembling a leaf.

**Distribution**. As yet known only from the Kamchatka Peninsula, Russia.

#### *Pseudoplanothidium minutum* Tseplik, Glushchenko, Genkal, Maltsev et Kulikovskiy sp. nov. (Fig 11).

**Holotype**. Slide no. 08309 (represented here by Fig 11C), deposited in the Herbarium of K.A. Timiryazev Institute of Plant Physiology, Russian Academy of Sciences (HD), Moscow, Russia, prepared from oxidized culture strain CBMCkam478.

**Isotype**. Slide no. 08309b, Herbarium of Main Botanical Garden, Russian Academy of Science (MHA), Moscow, Russia.

**Reference strain**. CBMCkam478 from the Culture and Barcode Collection of Microalgae and Cyanobacteria “Algabank” (CBМC), isolated from sample 166.

**Type locality**. Russia, Yuzhnaya Bay, sample 166, phytoplankton (N 52.952.434 E 158.687.17), *leg*. M.S. Kulikovskiy & N.V. Lobus, 17.07.2021.

**Description**. **LM**. Valves elliptic-lanceolate, with rounded to slightly protracted ends. Length 7.4–8.3 μm, width 3.5–4.0 μm. Raphe filiform, straight. Axial area on the raphe valve narrow linear, central area absent. Axial area on the rapheless valve also narrow linear to linear-lanceolate, central area represented by a pair of central striae spaced slightly wider than the rest on one side of the valve. Striae on both valves parallel to weakly radiate in the central part of the valve, becoming more radiate towards the ends, 16–19 in 10 μm.

**SEM**. External central raphe ends straight, expanded, distal ends turned to one side and extended onto the mantle. Internal central raphe ends turned slightly to opposite sides, distal ends terminate in faint helictoglossae. Striae multiseriate on both valves, consisting of 2–3 rows of circular areolae; the central row of areolae in a stria is typically smaller than the other two, which is especially noticeable on the rapheless valve. The axial area on the rapheless valve is slightly depressed. Internally, the interstriae are distinctly raised.

**Sequence data**. Partial 18S rDNA gene sequence comprising V4 domain sequence (GenBank accession number PX412396) and partial *rbc*L sequence (GenBank accession number PX418661) for the strain CBMCkam478.

**Etymology**. The epithet refers to the small valve size of the new species.

**Distribution**. As yet known only from type locality.

#### New combinations in the genus Pseudoplanothidium gen. nov.

##### *Pseudoplanothidium asymmetricum* (Gogorev et Lange) Tseplik, Glushchenko, Genkal, Maltsev et Kulikovskiy comb. nov.

**Basionym*:*** *Planothidium asymmetricum* Gogorev et Lange 2015. Achnanthoid diatoms (Bacillariophyta) of the relict Lake Mogilnoye (Kildin Island, Barents Sea). Nov. Sist. Nizsh. Rast. 49: 15; pl. III, figs 13,15.

**Type**:—Russia, Barents Sea, Kildin Island, Lake Mogilnoye (G18—LE, Plate III, *13, 15*).

##### *Pseudoplanothidium audax* (E. Morales, Novais et M.L. García) Tseplik, Glushchenko, Genkal, Maltsev et Kulikovskiy comb. nov.

**Basionym:** *Pl*anothidium audax** E. Morales, Novais et M.L. García in Morales et al. 2021. *Planothidium audax* sp. nov. (Bacillariophyta, Achnanthidiaceae), a new diatom from temporary streams in southern Portugal. Phytotaxa 510 (3): 288, figs 1–26, 68–73.

**Type**:—Portugal, Alentejo region, Serpa Council, Limas Stream (holotype: BR-4663 = Figs 21, 22).

##### *Pseudoplanothidium australe* (Manguin) Tseplik, Glushchenko, Genkal, Maltsev et Kulikovskiy comb. et stat. nov.

**Basionym:** *Ac*hnanthes delicatula** var. *australis* Manguin in Bourrelly, Manguin 1954. Contribution à la flore algale d’eau douce des Iles Kerguelen. Mémoires de l’Institut Scientifique de Madagascar, Série B, Biologique Végétale 5: 20, pl. II (2): fig. 18.

**Type**:—Sub-Antarctic region, Îles Kerguelen (see Bourrelly & Manguin 1954).

**Synonyms:** *Planothidium australe* (Manguin) Le Cohu 2005, Arch. Hydrobiol. Suppl. 157 (Algol. Stud. 116): 90.

##### *Pseudoplanothidium australodelicatulum* (Van de Vijver, C.E.Wetzel et Ector) Tseplik, Glushchenko, Genkal, Maltsev et Kulikovskiy comb. nov.

**Basionym:** *P*lanothidium australodelicatulum** Van de Vijver, C.E.Wetzel et Ector 2018. Analysis of the type material of *Planothidium delicatulum* (Bacillariophyta) with the description of two new *Planothidium* species from the sub-Antarctic Region. Fottea 18 (2): 206, figs 47–71.

**Type**:—Sub-Antarctic region, Prince Edward Islands, Marion Island, Ship’s Cove (holotype: BR–4520, figs 47–71).

##### *Pseudoplanothidium bahlsii* (Potapova) Tseplik, Glushchenko, Genkal, Maltsev et Kulikovskiy comb. nov.

**Basionym:** *P*latessa bahlsii** Potapova 2012. New species and combinations in monoraphid diatoms (family Achnanthidiaceae) from North America. Diatom Research 27 (1–2): 38, figs 84–96.

**Type**:—USA, New Jersey, Bergen County, Masonicus Brook (holotype: ANSP GC58969, figs 92–93).

**Synonyms**: *P*lanothidium bahlsii** (Potapova) M.S. Kulikovskiy, A.M. Glushchenko et J.P. Kociolek in Kulikovskiy et al. 2020, Fottea 20 (1): 65.

##### *Pseudoplanothidium baicalopumilum* (Kulikovskiy et Lange-Bertalot) Tseplik, Glushchenko, Genkal, Maltsev et Kulikovskiy comb. nov.

**Basionym:** *Plan*othidium baicalopumilum** Kulikovskiy et Lange-Bertalot in Kulikovskiy et al. 2015. Lake Baikal: hotspot of endemic diatoms II. Iconographia Diatomologica 26: 58, pl. 67: figs 1–25.

**Type**:—Russia, Lake Baikal, Bolshoi Ushkaniy Island (holotype: IBIW 15645m = Fig. 67:1).

##### *Pseudoplanothidium baicalorhombicum* (Kulikovskiy et Lange-Bertalot) Tseplik, Glushchenko, Genkal, Maltsev et Kulikovskiy comb. nov.

**Basionym:** *Plan*othidium baicalorhombicum** Kulikovskiy et Lange-Bertalot in Kulikovskiy et al. 2015. Lake Baikal: hotspot of endemic diatoms II. Iconographia Diatomologica 26: 59, pl. 68: figs 1–18.

**Type**:—Russia, Lake Baikal, Bolshoi Ushkaniy Island (holotype: IBIW 15645m = Fig. 68:1).

##### *Pseudoplanothidium campechianum* (Hustedt) Tseplik, Glushchenko, Genkal, Maltsev et Kulikovskiy comb. nov.

**Basionym:** *Achnanthes campechiana* Hustedt 1952. Neue und wenig bekannte Diatomeen. IV. Botaniska Notiser 4: 389, figs 87–90.

**Type**:—Mexico, Campêche Bay (lectotype: BRM Zt3/72, see Simonsen 1987: 386).

**Synonyms**: *Planothidium campechianum* (Hustedt) Witkowski, Lange-Bertalot et Metzeltin 2000, Iconographia Diatomologica 7: 118, pl. 48: figs 3–9.

##### *Pseudoplanothidium daui* (Foged) Tseplik, Glushchenko, Genkal, Maltsev et Kulikovskiy comb. nov.

**Basionym:** *Achnanthes daui* Foged 1962. On the diatom flora in interglacial kieselguhr at Hollerup in East Jutland. Danmarks Geologiske Untersogelse, 2nd Series 84: 14, pl. 1: fig. 10.

**Type:—**Denmark, Hollerup (holotype: N. Foged 2205 (36), pl. 1: fig. 10).

**Synonyms**: *Achnantheiopsis daui* (Foged) Lange-Bertalot 1997, Arch. Protistenk. 148: 206, figs 18–20; *Planothidium daui* (Foged) Lange-Bertalot 1999, Iconographia Diatomologica 6: 275.

##### *Pseudoplanothidium delicatulum* (Kützing) Tseplik, Glushchenko, Genkal, Maltsev et Kulikovskiy comb. nov.

**Basionym:** *Achnanthidium delicatulum* Kützing 1844. Die Kieselschaligen Bacillarien oder Diatomeen. Nordhausen: zu finden bei W. Köhne. 75, pl. 3: fig. XXI.

**Type**:—Germany, East Frisian Islands, Wangerooge (lectotype: BR–4461, see Van de Vijver et al. 2018, Figs 1–2).

**Synonyms**: *Falcatella delicatula* (Kützing) Rabenhorst 1853: 46, pl.5 fig.4; *Achnanthes delicatula* (Kützing) Grunow in van Heurck 1880: pl. XXVII fig. 3; *Microneis delicatula* (Kützing) P.T. Cleve 1895, Kongliga Svenska Vetenskapsakademiens Handlingar Series 4 26 (2): 190; *Achnantheiopsis delicatula* (Kützing) Lange-Bertalot 1997, Arch. Protistenk. 148: 206, fig. 10; *Planothidium delicatulum* (Kützing) Round et Bukhtiyarova 1996, Diatom Research 11 (2): 353.

##### *Pseudoplanothidium densistriatum* (Van de Vijver et Beyens) Tseplik, Glushchenko, Genkal, Maltsev et Kulikovskiy comb. nov.

**Basionym:** *P*lanothidium densistriatum** Van de Vijver et Beyens in Van de Vijver et al. 2002. Freshwater diatoms from Île de la Possession (Crozet Archipelago, sub-Antarctica). Bibliotheca Diatomologica 46: 100, pl. 25, figs 1–17.

**Type**:—Sub-Antarctic region, Crozet Archipelago, Île de la Possession, Crique de Noёl (holotype: CAS-220057, pl. 25, figs 1–17).

##### *Pseudoplanothidium deperditum* (Giffen) Tseplik, Glushchenko, Genkal, Maltsev et Kulikovskiy comb. nov.

**Basionym:** *Cocconeis deperdita* Giffen 1975. An account of the littoral diatoms from Langebaan, Saldanha Bay, Cape Province, South Africa. Botanica Marina 18: 78, figs 26–28.

**Type**:—South Africa, Cape Province, Saldanha Bay, Langebaan (holotype: 632 Giffen Collection, figs 26–28).

**Synonyms**: *Planothidium deperditum* (Giffen) Witkowski, Lange-Bertalot et Metzeltin 2000, Iconographia Diatomologica 7: 119, pl. 36: figs 31–33; pl. 49: figs 12,13.

##### *Pseudoplanothidium diplopunctatum* (Simonsen) Tseplik, Glushchenko, Genkal, Maltsev et Kulikovskiy comb. nov.

**Basionym:** *A*chnanthes diplopunctata** Simonsen in Hinz et al. 2012. Validation of 42 names of diatom taxa from the Baltic Sea. Diatom Research 27 (2): 82.

**Type:**—Baltic Sea, Boknis-Eck (holotype: BRM SIM5/19, see Kusber et al. 2023).

**Synonyms:** *Planothidium diplopunctatum* (Simonsen) Witkowski et Lange-Bertalot in Kusber et al. 2023, Notulae Algarum 287: 2.

##### *Pseudoplanothidium dispar* (P.T. Cleve) Tseplik, Glushchenko, Genkal, Maltsev et Kulikovskiy comb. nov.

**Basionym:** *Achnanthes dispar* P.T. Cleve 1891. The diatoms of Finland. Acta Societatia pro Fauna et Flora Fennica 8 (2): 52, pl. 3: figs 2, 3.

**Type**:—Finland, Gulf of Bothnia near Torneå (see Cleve 1891, pl. 3: figs 2, 3).

**Synonyms:** *Actinoneis dispar* (P.T. Cleve) P.T. Cleve 1895, Kongliga Svenska Vetenskaps-Akademiens Handlingar 27 (3): 186; *Achnanthes dispar* var. *angulata* Hustedt 1930: 204, fig. 296b; *Planothidium dispar* (P.T. Cleve) Witkowski, Lange-Bertalot et Metzeltin 2000, Iconographia Diatomologica 7: 120, pl. 49: figs 16–19.

##### *Pseudoplanothidium distinctum* (Messikommer) Tseplik, Glushchenko, Genkal, Maltsev et Kulikovskiy comb. nov.

**Basionym:** *Ac*hnanthes distincta** Messikommer 1954. Beiträge zur Kenntnis der Algenflora des Urner Reusstales (Centralschweiz). Hydrobiologie 6: 32, pl. 1: figs 2a,b.

**Type:—**Switzerland, Lauteres Seeli (lectotype: BP 2210!, see Buczkó et al. 2013, fig. 5).

**Synonyms**: *Achnantheiopsis distincta* (Messikommer) Lange-Bertalot 1997, Arch. Protistenk. 148: 206; *Planothidium distinctum* (Messikommer) Lange-Bertalot 1999, Iconographia Diatomologica 6: 275.

##### *Pseudoplanothidium engelbrechtii* (Cholnoky) Tseplik, Glushchenko, Genkal, Maltsev et Kulikovskiy comb. nov.

**Basionym:** *A*chnanthes engelbrechtii** Cholnoky 1955. Hydrobiologische Untersuchungen in Transvaal. I. Vergelichung der herbstlichen Algengemeinschaften in Rayton-vlei und Leeufontein. Hydrobiologia 7: 16, figs 1–8.

**Type:**—South Africa, Cape Province, Jakkals, Olifantsriver (lectotype: CSIR 326/6504, see Compère & Van de Vijver 2009).

**Synonyms**: *Achnanthes delicatula* subsp. *engelbrechtii* (Cholnoky) Lange-Bertalot in Krammer, Lange-Bertalot, 1991: 72; *Achnantheiopsis engelbrechtii* (Cholnoky) Lange-Bertalot 1997, Arch. Protistenk. 148: 206; *Planothidium engelbrechtii* (Cholnoky) Round et Bukhtiyarova 1996, Diatom Research 11 (2): 353.

##### *Pseudoplanothidium galaicum* (I. Álvarez-Blanco et S. Blanco) Tseplik, Glushchenko, Genkal, Maltsev et Kulikovskiy comb. nov.

**Basionym:** *Planothidium galaicum* I. Álvarez-Blanco et S. Blanco 2013. *Planothidium galaicum* sp. nov. (Bacillariophyta, Achnanthidiaceae), a new diatom species from Galician coast, Spain. Phytotaxa 151 (1): 45, figs 2–25.

**Type**:—Spain, Muxía coast (holotype: LEB! 024, figs 2–25).

##### *Pseudoplanothidium granum* (M.H. Hohn et Hellerman) Tseplik, Glushchenko, Genkal, Maltsev et Kulikovskiy comb. nov.

**Basionym:** *Achnanthes grana* M.H. Hohn et Hellerman 1963. The taxonomy and structure of diatom populations from three eastern North American rivers using three sampling methods. Transactions of the American Microscopical Society 82 (3): 274, pl. 2: figs 9, 10.

**Type:—**Canada, Ontario, La Vase River (holotype: G.C. 44450, pl. 2: figs 9, 10).

**Synonyms**: *Achnantheiopsis grana* (M.H. Hohn et Hellerman) Lange-Bertalot 1997, Arch. Protistenk. 148: 207, figs 16–17; *Planothidium granum* (M.H.Hohn et Hellerman) Lange-Bertalot 1999, Iconographia Diatomologica 6: 276.

##### *Pseudoplanothidium hauckianum* (Grunow) Tseplik, Glushchenko, Genkal, Maltsev et Kulikovskiy comb. nov.

**Basionym:** *Achnanthes hauckiana* Grunow in P.T. Cleve & Grunow 1880. Beiträge zur Kenntniss der arctischen Diatomeen. Kongliga Svenska Vetenskaps-Akademiens Handlingar 17 (2): 21.

**Type:**—Italy, Reka spring near Trieste (holotype: 1238 Grunow Collection, see Kulaš et al. 2020, figs 1–63).

**Synonyms**: *Microneis hauckiana* (Grunow) P.T. Cleve 1895, Kongliga Svenska Vetenskapsak-Akademiens Handlingar 27 (3): 190; *Achnanthes delicatula* subsp. *hauckiana* (Grunow) Lange-Bertalot et Ruppel 1980, Algological Studies/Archiv für Hydrobiologie, Supplement Volumes 26: 6, figs 1–20, 268 a–c, 330, 331; *Achnanthidium hauckianum* (Grunow) D.B. Czarnecki 1994, Journal of the Iowa Academy of Science 101 (3–4): 96; *Achnantheiopsis hauckiana* (Grunow) Lange-Bertalot 1997, Arch. Protistenk. 148: 207; *Planothidium hauckianum* (Grunow) Bukhtiyarova 1999: 44.

##### *Pseudoplanothidium heidenii* (P.Schultz) Tseplik, Glushchenko, Genkal, Maltsev et Kulikovskiy comb. nov.

**Basionym:** *Achnanthes heidenii* P. Schulz 1926. Die Kieselalgen der Danziger Bucht mit Einschluss derjenigen aus glazialen und postglazialen Sedimenten. Botanische Archiv 13 (3–4): 190, fig. 36.

**Type:**—Poland, Gulf of Gdansk, Hel Peninsula (see Witkowski et al. 2000: 120).

**Synonyms:** *Planothidium heidenii* (P. Schultz) Witkowski, Lange-Bertalot et Metzeltin 2000, Iconographia Diatomologica 7: 120, pl. 51: figs 21, 22.

##### *Pseudoplanothidium holstii* (P.T. Cleve) Tseplik, Glushchenko, Genkal, Maltsev et Kulikovskiy comb. nov.

**Basionym:** *Ac*hnanthes holstii** Cleve 1882 (‘1881’). Färskvattens-Diatomacéer från Grönland och Argentinska Republiken. Öfversigt af Kongliga Svenska Vetenskaps-Akademiens Förhandlingar 38 (10): 13, pl. XVI: figs 6, 7.

**Type:—**Greenland, Kornak (see Cleve 1882: 13, pl. XVI: figs 6, 7).

**Synonyms:** *Achnantheiopsis holstii* (Cleve) Lange-Bertalot 1997, Arch. Protistenk. 148: 207; *Planothidium holstii* (Cleve) Lange-Bertalot 1999, Iconographia Diatomologica 6: 277.

##### *Pseudoplanothidium iberense* (L. Rovira et Witkowski) Tseplik, Glushchenko, Genkal, Maltsev et Kulikovskiy comb. nov.

**Basionym:** *P*lanothidium iberense** L. Rovira et Witkowski in Rovira et al. 2011. *Planothidium iberense* sp. nov., a new brackish diatom of the Ebro Estuary, northeast Spain. Diatom Research 26 (1): 100, figs 2–24.

**Type**:—Spain, Ebro River Estuary (holotype: 14825, collection A. Witkowski = Fig. 2).

##### *Pseudoplanothidium jan-marcinii* (Witkowski, Metzeltin et Lange-Bertalot) Tseplik, Glushchenko, Genkal, Maltsev et Kulikovskiy comb. nov.

**Basionym:** *Achnanthes jan-marcinii* Witkowski, Metzeltin et Lange-Bertalot in Metzeltin, Witkowski 1996. Diatomeen der Bären-Insel. Süsswasser- und marine Arten. Iconographia Diatomologica 4: 2, pl. 50: figs. 5–9, 22, 23; pl. 75: figs. 4, 9.

**Type**:—Norway, Svalbard, Bear Island (holotype: Bären-Insel No. 40−2, fig. 50: 5–9).

**Synonyms**: *P*lanothidium jan-marcinii** (Witkowski, Metzeltin et Lange-Bertalot) Witkowski, Lange-Bertalot et Metzeltin 2000, Iconographia Diatomologica 7: 121, pl. 49: figs 29–33.

##### *Pseudoplanothidium juandenovense* (Riaux-Gobin et Witkowski) Tseplik, Glushchenko, Genkal, Maltsev et Kulikovskiy comb. nov.

**Basionym:** *P*lanothidium juandenovense** Riaux-Gobin et Witkowski in Riaux-Gobin et al. 2018. *Planothidium juandenovense* sp. nov. (Bacillariophyta) from Juan de Nova (Scattered Islands, Mozambique Channel) and other tropical environments: a new addition to the *Planothidium delicatulum* complex. Fottea 18 (1): 108, figs. 1–20.

**Type**:—Juan de Nova Island (holotype: BM 101 810 = Fig. 4).

##### *Pseudoplanothidium kaetherobertianum* (Van de Vijver et Bosak) Tseplik, Glushchenko, Genkal, Maltsev et Kulikovskiy comb. nov.

**Basionym:** *P*lanothidium kaetherobertianum** Van de Vijver et Bosak 2019. *Planothidium kaetherobertianum*, a new marine diatom (Bacillariophyta) species from the Adriatic Sea. Phytotaxa 425 (2): 106, figs 1–47.

**Type**:—Croatia, Adriatic Sea, Pula (holotype BR! 4570, figs 1–47).

##### *Pseudoplanothidium lacustre* (I. Álvarez-Blanco, C. Cejudo-Figueiras et S. Blanco) Tseplik, Glushchenko, Genkal, Maltsev et Kulikovskiy comb. nov.

**Basionym:** *P*lanothidium lacustre** I. Álvarez-Blanco, C. Cejudo-Figueiras et S. Blanco in Blanco et al. 2013. The diatom flora in temporary ponds of Doñana National Park (southwest Spain): five new taxa. Nordic Journal of Botany 31: 491, figs 13–28.

**Type**:—Spain, Iberian Peninsula, Navazo del Toro pond (holotype: LEB 012, figs 13–28).

##### *Pseudoplanothidium lilianeanum* (Van de Vijver) Tseplik, Glushchenko, Genkal, Maltsev et Kulikovskiy comb. nov.

**Basionym:** *P*lanothidium lilianeanum** Van de Vijver in Van de Vijver et al. 2018. Analysis of the type material of *Planothidium delicatulum* (Bacillariophyta) with the description of two new *Planothidium* species from the sub-Antarctic Region. Fottea 18 (2): 203, figs 29–46.

**Type**:—Sub-Antarctic region, Iles Kerguelen, Grand–Terre, Val Studer (holotype: BR–4520, figs 29–46).

##### *Pseudoplanothidium lilljeborgei* (Grunow) Tseplik, Glushchenko, Genkal, Maltsev et Kulikovskiy comb. nov.

**Basionym:** *A*chnanthes lilljeborgei** Grunow 1881. Über die Arten der Gattung *Grammatophora* mit Bezug auf die Tafeln LIII und LIIIB in Van Heurck’s Synopsis der belgischen Diatomeen. Botanisches Centralblatt 7: 68.

**Type**:—Norway, Grip (see Cleve & Möller, Diat. Nr. 311).

**Synonyms**: *Planothidium lilljeborgei* (Grunow) Witkowski, Lange-Bertalot et Metzeltin 2000, Iconographia Diatomologica 7: 121, pl. 49: fig. 1; pl. 51: figs 27–29.

##### *Pseudoplanothidium linkei* (Hustedt) Tseplik, Glushchenko, Genkal, Maltsev et Kulikovskiy comb. nov.

**Basionym:** *Achnanthes linkei* Hustedt 1939. Die Diatomeenflora des Küstengebietes der Nordsee vom Dollart bis zur Elbemündung. I. Die Diatomeenflora in den Sedimenten der unteren Ems sowie auf den Watten in der Leybucht, des Memmert und bei der Insel Juist. Adhandlungen des Naturwissenschaftlichen Verein zu Bremen 31(2/3): 607, figs 28–32.

**Type**:—Germany, Wangerooge (lectotype: BRM 05/60, see Simonsen 1987: 253, pl. 377: figs 6-9).

**Synonyms**: *Achnanthes linkei* var. *rostrata* Schulz 1926; *Achnanthes haynaldii* var. *linkei* (Hustedt) A.Cleve 1953, Kungliga Svenska Vetenskapsakademiens Handlingar, ser. IV 4 (5): 27, fig. 528 k,l; *Achnanthes pictii* J.R.Carter 1963; *Achnantheiopsis linkei* (Hustedt) Lange-Bertalot 1997, Arch. Protistenk. 148: 207; *Planothidium linkei* (Hustedt) Lange-Bertalot 1999, Iconographia Diatomologica 6: 277.

##### *Pseudoplanothidium marginostriatum* (Van de Vijver et Beyens) Tseplik, Glushchenko, Genkal, Maltsev et Kulikovskiy comb. nov.

**Basionym:** *Pla*nothidium marginostriatum** Van de Vijver et Beyens in Van de Vijver et al. 2002. Freshwater diatoms from Île de la Possession (Crozet Archipelago, sub-Antarctica). Bibliotheca Diatomologica 46: 101, pl. 26: 1–17.

**Type**:—Sub-Antarctic region, Crozet Archipelago, Île de la Possession, Crique de Noёl (holotype: CAS-220092, pl. 26: 1–17).

##### *Pseudoplanothidium marinum* (Hustedt ex Simonsen) Tseplik, Glushchenko, Genkal, Maltsev et Kulikovskiy comb. nov.

**Basionym:** *A*chnanthes marina** Hustedt ex Simonsen 1987. Atlas and catalogue of the diatom types of Friedrich Hustedt. Berlin & Stuttgart: J. Cramer in der Gebrüder Borntraeger Velagsbuchhandlung. P. 231, pl. 337: figs 1–6.

**Type:**—Spain, Balearic Islands (holotype: N18/90, see Simonsen 1987: 231, pl. 337: 1–6).

**Synonyms:** *P*lanothidium marinum** (Hustedt ex Simonsen) Lange-Bertalot 1999, Iconographia Diatomologica 6: 277.

##### *Pseudoplanothidium mathurinense* (Riaux-Gobin et Al-Handal) Tseplik, Glushchenko, Genkal, Maltsev et Kulikovskiy comb. nov.

**Basionym:** *Planothidium mathurinense* Riaux-Gobin et Al-Handal in Riaux-Gobin et al. 2012. SEM survey of some small-sized *Planothidium* (Bacillariophyta) from coral sand off Mascarenes (Western Indian Ocean). Nova Hedwigia Beiheft 141: 302, figs 2–5, 6–8–8, 26–38–38.

**Type**:—Western Indian Ocean, Rodrigues Island, Port Mathurin (holotype: USR 3278 = Fig. 28).

##### *Pseudoplanothidium minutissimum* (Krasske) Tseplik, Glushchenko, Genkal, Maltsev et Kulikovskiy comb. et stat. nov.

**Basionym:** *Achnanthes lanceolata* var. *minutissima* Krasske 1938. Beiträge zur Kenntnis der Diatomeen-Vegetation von Island und Spitzsbergen. Archiv für Hydrobiologie 33: 513.

**Type**:—Iceland (lectotype: C II 77, see Lange-Bertalot et al. 1996: 32).

**Synonyms**: *Achnantheiopsis minutissima* (Krasske) Lange-Bertalot 1997, Arch. Protistenk. 148: 207; *Planothidium minutissimum* (Krasske) Lange-Bertalot 2006, Iconographia Diatomologica 6: 278.

##### *Pseudoplanothidium neglectum* (Lange-Bertalot et U. Rumrich) Tseplik, Glushchenko, Genkal, Maltsev et Kulikovskiy comb. nov.

**Basionym:** *P*lanothidium neglectum** Lange-Bertalot et U. Rumrich in Rumrich et al. 2000. Diatomeen der Anden von Venezuela bis Patagonien/Feuerland und zwei weitere Beiträge. Diatoms of the Andes from Venezuela to Patagonia/Tierra del Fuego and two additional contributions. Iconographia Diatomologica 9: 213, pl. 30, figs 7–10.

**Type:**—Chile, Altiplano, Río Lauca (holotype: AmS-Ch 60, fig. 30: 7–10).

##### *Pseudoplanothidium oculatum* (Hustedt) Tseplik, Glushchenko, Genkal, Maltsev et Kulikovskiy comb. nov.

**Basionym:** *Achnanthes oculata* Hustedt 1952. Neue und wenig bekannte Diatomeen. IV. Botaniska Notiser 4: 390, figs 91–94.

**Type**:—Mexico, Campêche Bay (lectotype: BRM Zt3/72, see Simonsen 1987: 386).

**Synonyms**: *Planothidium oculatum* (Hustedt) Witkowski, Lange-Bertalot et Metzeltin 2000, Iconographia Diatomologica 7: 122, pl. 49: figs 40, 41.

##### *Pseudoplanothidium pericavum* (J.R. Carter) Tseplik, Glushchenko, Genkal, Maltsev et Kulikovskiy comb. nov.

**Basionym:** *Achnanthes pericava* J.R. Carter 1966. Some freshwater diatoms of Tristan da Cunha and Gough Island. Nova Hedwigia 11: 447, pl. 60 (1): figs 5–8.

**Type:**—Tristan da Cunha (holotype: ВМ 77593, see Carter 1966: 447).

**Synonyms:** *Achnantheiopsis pericava* (J.R. Carter) Lange-Bertalot 1997, Arch. Protistenk. 148: 207; *Planothidium pericavum* (J.R. Carter) Lange-Bertalot 1999, Iconographia Diatomologica 6: 278.

##### *Pseudoplanothidium polare* (Østrup) Tseplik, Glushchenko, Genkal, Maltsev et Kulikovskiy comb. nov.

**Basionym:** *Achnanthes polaris* Østrup 1895. Marine diatoméer fra Østgrønland. Meddelelser om Grønland, Kjøbenhavn 18: 85, pl. 7: fig. 86.

**Type**:—Greenland (holotype: Coll. Østrup im Bot. Mus. Copenhagen 533, see Krammer & Lange-Bertalot 1989: 318).

**Synonyms**: *P*lanothidium polare** (Østrup) Witkowski, Lange-Bertalot et Metzeltin 2000, Iconographia Diatomologica 7: 123, pl. 47: 1–4; pl. 49: figs 37–39.

##### *Pseudoplanothidium pumilum* (Bąk et Lange-Bertalot) Tseplik, Glushchenko, Genkal, Maltsev et Kulikovskiy comb. nov.

**Basionym**: *Planothidium pumilum* Bąk et Lange-Bertalot 2015. Four small-celled *Planothidium* species from Central Europe proposed as new to science. Oceanological and Hydrobiological Studies 43 (4): 350, fig. 4 a–z; pl. 5 a–e; pl. 6 a–d.

**Type**:—Poland, Baltic Sea, Świna Strait near the village of Karsibór (holotype: SZCZ 9165 = Fig. 4: a, b).

##### *Pseudoplanothidium quadripunctatum* (Oppenheim) Tseplik, Glushchenko, Genkal, Maltsev et Kulikovskiy comb. nov.

**Basionym:** *Achnanthes quadripunctata* Oppenheim 1994. Taxonomic studies of *Achnanthes* (Bacillariophyta) in freshwater maritime antarctic lakes. Canadian Journal of Botany, 72: 1742, figs 26–29, 63–66–66.

**Type**:—South Shetland Islands, Signy Island, Light Lake, Twisted Lake (holotype: BM 92689, Figs 26–29, 63–66–66).

**Synonyms**: *Planothidium quadripunctatum* (Oppenheim) Sabbe 2003, Antarctic Science 15 (2): 241, figs 13, 14, 73.

##### *Pseudoplanothidium quarnerense* (Grunow) Tseplik, Glushchenko, Genkal, Maltsev et Kulikovskiy comb. nov.

**Basionym:** *Rhaphoneis quarnerensis* Grunow 1862. Die österreichischen Diatomaceen nebst Anschluss einiger neuen Arten von andern Lokalitäten und einer kritischen Uebersicht der bisher bekannten Gattungen und Arten. Verhandlungen der kaiserlich-königlichen zoologisch-botanischen Gesellschaft in Wien 12: 381, pl. 4.7: fig. 24.

**Type**:—Adriatic Sea (see Witkowski et al. 2000: 123).

**Synonyms**: *Cocconeis quarnerensis* (Grunow) A.W.F. Schmidt 1874, Jahresbericht der Kommission zur Untersuchung der Deutsch Meer 2: 93, pl. 3: fig. 16; *Planothidium quarnerense* (Grunow) Witkowski, Lange-Bertalot et Metzelin 2000, Iconographia Diatomologica 7: 123, pl. 55: figs 2–7.

##### *Pseudoplanothidium renei* (Lange-Bertalot et Rol. Schmidt) Tseplik, Glushchenko, Genkal, Maltsev et Kulikovskiy comb. nov.

**Basionym:** *A*chnanthes renei** Lange-Bertalot et Rol. Schmidt in Schmidt et al. 1990. Holocene diatom flora and stratigraphy from sediment cores of two Antarctic lakes (King George Island). Journal of Paleolimnology 3: 64, figs 6o–t.

**Type**:—Antarctica, King George Island, Fildes Peninsula, Monsee (holotype: Am-S91, figs 6o–t).

**Synonyms**: *Pla*nothidium renei** (Lange-Bertalot et Rol. Schmidt) Van de Vijver in Van de Vijver et al. 2002, Bibliotheca Diatomologica 46: 102.

##### *Pseudoplanothidium robustum* (Hustedt) Tseplik, Glushchenko, Genkal, Maltsev et Kulikovskiy comb. et stat. nov.

**Basionym:** *A*chnanthes delicatula** var*. robusta* Hustedt 1934. Die Diatomeenflora von Poggenpohls Moor bei Dötlingen in Oldenburg. Abhandlungen und Vorträgen der Bremer Wissenschaftlichen Gessellschaft 8/9: 378, figs 1, 2.

**Type**:—Germany, Oldenburg, near Dotlingen, Poggenpohls Moor (holotype: Ma 1/50, see Lange-Bertalot & Krammer 1989: 326).

**Synonyms**: *Ac*hnantheiopsis robusta** (Hustedt) Lange-Bertalot 1997, Arch. Protistenk. 148: 208, fig. 21; *Planothidium robustum* (Hustedt) Lange-Bertalot 1999, Iconographia Diatomologica 6: 279.

##### *Pseudoplanothidium rodriguense* (Riaux-Gobin et Compère) Tseplik, Glushchenko, Genkal, Maltsev et Kulikovskiy comb. nov.

**Basionym**: *Planothidium rodriguense* Riaux-Gobin et Compère in Riaux-Gobin et al. 2012. SEM survey of some small-sized *Planothidium* (Bacillariophyta) from coral sand off Mascarenes (Western Indian Ocean). Nova Hedwigia Beiheft 141: 297, figs 1, 13–25–25.

**Type**:—Western Indian Ocean, Rodrigues Island, Port Mathurin (holotype: USR 3278 = Fig. 22).

##### *Pseudoplanothidium schwabei* (Krasske) Tseplik, Glushchenko, Genkal, Maltsev et Kulikovskiy comb. nov.

**Basionym**: *A*chnanthes schwabei** Krasske 1939. Zur Kieselalgenflora Südchiles. Archiv für Hydrobiologie und Planktonkunde, Stuttgart 35 (3): 371, pl. 11: figs 12–14.

**Type**:—Chile, shores of Puyuhuapi-Fjordes (lectotype: Coll. Krasske D III 246, see Lange-Bertalot et al. 1996: 214).

**Synonyms:** *Planothidium schwabei* (Krasske) Lange-Bertalot in Rumrich et al. 2000, Iconographia Diatomologica 9: 216.

##### *Pseudoplanothidium septentrionale* (Østrup) Tseplik, Glushchenko, Genkal, Maltsev et Kulikovskiy comb. nov.

**Basionym:** *Achnanthes septentrionalis* Østrup 1910. Danske Diatoméer med 5 tavler et Engelsk résumé. Udgivet paa Carlsbergfondets bekostning. Copenhagen: C.A. Reitzel Boghandel Bianco Lunos Bogtrykker: 215, fig 13: 27.

**Type**:—Greenland (lectotype: Coll. Østrup, Bot. Mus. Copenhagen 4641, see Lange-Bertalot & Krammer 1989: 330).

**Synonyms**: *Planothidium septentrionale* (Østrup) Round et Bukhtiyarova 1996, Diatom Research 11 (2): 353.

##### *Pseudoplanothidium suncheonmanense* (Jahn et Zimmermann) Tseplik, Glushchenko, Genkal, Maltsev et Kulikovskiy comb. nov.

**Basionym:** *Planothidium suncheonmanense* Jahn et Zimmermann in Jahn et al. 2017. *Planothidium lanceolatum* and *Planothidium frequentissimum* reinvestigated with molecular methods and morphology: four new species and the taxonomic importance of the sinus and cavum. Diatom Research 32 (1): 101.

**Type**:—Korea, ChollaNamdo, SunCheonMan (holotype: B 40 0041403, strain Ko0408 = Fig. 376).

##### *Pseudoplanothidium tenerum* (Hustedt) Tseplik, Glushchenko, Genkal, Maltsev et Kulikovskiy comb. nov.

**Basionym:** *Achnanthes tenera* Hustedt 1955. Marine littoral diatoms of Beaufort, North Carolina. Bulletin Duke University Marine Station 6: 17, pl. 5: figs 22–25.

**Type**:—USA, North Carolina, Beaufort (holotype: BRM Ztl/96, see Simonsen 1987: 407).

**Synonyms:** *Planothidium tenerum* (Hustedt) Compère et Riaux-Gobin in Riaux-Gobin et al. 2012, Nova Hedwigia Beiheft 141: 311.

##### *Pseudoplanothidium vanheurckii* (Grunow in Van Heurck) Tseplik, Glushchenko, Genkal, Maltsev et Kulikovskiy comb. nov.

**Basionym:** *Rhoicosphenia vanheurckii* Grunow in Van Heurck 1880. Synopsis des Diatomées de Belgique Atlas. Anvers: Ducaju et Cie. Pl. 26: figs 5–9.

**Type**:—Belgium, Brussels (lectotype: BR V-10-C12, see Thomas et al. 2015, figs 3–20).

**Synonyms**: *Planothidium vanheurckii* (Grunow in Van Heurck) E.W.Thomas, Van de Vijver et Kociolek 2015, Diatom Research 30 (4): 339.

##### *Pseudoplanothidium werumianum* (Lange-Bertalot et Bąk) Tseplik, Glushchenko, Genkal, Maltsev et Kulikovskiy comb. nov.

**Basionym:** *Planothidium werumianum* Lange-Bertalot et Bąk 2015. Four small-celled *Planothidium* species from Central Europe proposed as new to science. Oceanological and Hydrobiological Studies 43 (4): 347, figs 1: a–z, 2: a–e, 3: a–e.

**Type**:—Germany, Ihner Bach Mündung close to the village of Niedaltdorf (holotype: FR 190 Mosel and Nfl. = Fig. 1:c, d).

##### *Pseudoplanothidium wetzelectorianum* (Kopalová, Zidarova et Van de Vijver) Tseplik, Glushchenko, Genkal, Maltsev et Kulikovskiy comb. nov.

**Basionym:** *Pl*anothidium wetzelectorianum** Kopalová, Zidarova et Van de Vijver 2016. Four new monoraphid diatom species (Bacillariophyta, Achnanthaceae) from the Maritime Antarctic Region. European Journal of Taxonomy 217: 7, figs 25–57.

**Type**:—Antarctica, James Ross Island, Monolith Lake (holotype: BR–4437, Figs 25–57).

## Discussion

### *Planothidium* s. str.

In the original diagnosis of the genus *Planothidium*, Round and Bukhtiyarova [3] note that the striae on the rapheless valve may be continuous (*delicatula*-type) or interrupted by a horseshoe-like depression that may or may not be capped (*lanceolata*-type). *P. lanceolatum*, which has been designated as the type species of the genus, has a horseshoe-shaped structure on the rapheless valve in the form of a sinus. Therefore we suggest that *Planothidium* s.str. from now on include only species with a sinus, which is a unique structure that is not observed in any other monoraphid genera. This decision is further supported by molecular data that show species with a sinus clustered together in a distinct clade.

Additional distinguishing features of *Planothidium* include bi- to multiseriate striae on both valves (with up to 5 rows of areolae) [21] and terminal raphe fissures that are bent in the same direction [3]. Both of these are considered important features for distinguishing monoraphid taxa on genus level that are consistent with molecular data [21,22]. While unilaterally bent terminal raphe fissures are present in quite a lot of monoraphid genera, multiseriate striae are more rare. For example, *Haloroundia* Diaz et Maidana has triseriate striae on both raphe and rapheless valves [23]; *Xenobennettella* also has triseriate striae on both valves that become loose areolae towards the central part of the valve [24]. The recently described genus *Vallithidium* Nienow et Prasad is characterised by triseriate striae on the raphe valve and biseriate striae on the rapheless valve [25]. *Platebaikalia* Kulikovskiy, Glushchenko, Genkal et Kociolek is the most similar to *Planothidium* in terms of striae structure since it has multiseriate striae on the raphe valve and biseriate striae with tendency for multiseriate on the rapheless valve; however, the multiseriate striae in *Platebaikalia* contain up to 7 rows of areolae per stria, which differentiates it from *Planothidium* where striae only contain up to 5 rows of areolae [21]. As mentioned earlier, none of these genera have a sinus on the rapheless valve, which makes *Planothidium* quite distinctive among monoraphid genera.

### *Paraplanothidium* gen. nov.

The new genus *Paraplanothidium* gen. nov. is separated from *Planothidium* on the basis of possessing a horseshoe-shaped structure on the rapheless valve in the form of a cavum, i.e., covered internally with a thin flap of silica. Several other monoraphid genera possess a cavum as well, namely *Gliwiczia*, *Skabitschewskia*, *Planoplatessa* and *Xenobennettella*; however, it has been suggested previously that they are not closely related to each other [10]. *Gliwiczia* is unique among the cavum-bearing genera because it has a cavum on both raphe and rapheless valves [26], while in the other genera a cavum is present only on the rapheless valve. The shape of the cavum also varies, for example, in *Xenobennettella* it is long and narrow, with parallel sides and a small rounded opening [24], while *Planoplatessa* has a very wide cavum with a wide opening [27].

Other morphological features that can differentiate *Paraplanothidium* gen. nov. from other cavum-bearing genera include the structure of striae, which can be uni-, bi- or multiseriate, the shape of terminal raphe fissures, and the structure of central and axial areas. *Paraplanothidium* gen. nov. has multiseriate striae on both valves, formed by 2–6 rows of areolae per stria, which is characteristic for the *Planothidium* s.l. group. Other genera considered in this discussion do not share this feature: *Planoplatessa* and *Gliwiczia* are characterized by uniseriate striae on both valves, *Skabitschewskia* has uniseriate striae on the raphe valve and biseriate striae on the rapheless valve, and *Xenobennettella* possesses triseriate striae that become composed of loosely spread areolae towards the central part of the valve. The external distal raphe ends also vary between these genera; in *Xenobennettella* they are bent to one side and end on the valve mantle, same as in the new genus, while in *Gliwiczia* and *Skabitschewskia* they are bent to opposite sides and in *Planoplatessa* they are straight and end on the valve face. As for the structure of the central and axial areas, *Gliwiczia* has a prominent stauros on both valves which is not characteristic for *Paraplanothidium* gen nov.; *Xenobennettella* has a large area with loosely arranged areolae on the raphe valve that occupies most of the valve face and a one-sided central area on the rapheless valve; in *Skabitschewskia* this feature is variable. Generally the combination of the above mentioned features allows to delineate *Paraplanothidium* gen. nov. from other genera with confidence.

The new species descibed in this study, *Paraplanothidium laevis* sp. nov., is morphologically quite similar to two other species described from the Kamchatka Peninsula, *P. tyushovii* Tseplik, Glushchenko, Maltsev et Kulikovskiy and *P. dyakonovii* Tseplik, Glushchenko, Maltsev et Kulikovskiy [28]; in fact, these species can easily be confused when viewed in LM. However, careful examination and especially use of SEM reveals some subtle morphological differences that point to the fact that these strains represent independent species. In *P. laevis* sp. nov. the central area on the raphe valve is mostly absent or indistinct, while *P. tyushovii* and *P. dyakonovii* possess a small but prominent central area that is rectangular in *P. tyushovii* and elliptical or rounded in *P. dyakonovii*. *P. tyushovii* and *P. dyakonovii* both have shortened striae in the central part of the rapheless valve opposite the cavum, *P. laevis* sp. nov. does not. The ornamentation of the rapheless valve is also a differentiating feature [8]; *P. tyushovii* and *P. dyakonovii* have faint round depressions all along the axial area, while the valves of *P. laevis* sp. nov. are smooth. According to molecular data, strains that we assigned to *P. tyushovii*, *P. dyakonovii* and *P. laevis* sp. nov. belong to different subclades within the large clade, which further supports our decision to separate them into independent taxa.

*P. laevis* sp. nov. can also be compared to such known species as *P. frequentissimum*, *P. victori* and *P. naradoense*. These species can be distinguished from the new species by valve outline and shape of the valve ends, as well as by the presence of a pronounced central area on the raphe valve, while in *P. laevis* sp. nov. it is mostly absent. Interestingly, *P. laevis* sp. nov. does not seem to have any kind of ornamentation on the rapheless valve; Wetzel et al. [8] studied many common cavum-bearing *Planothidium* s.l. species and only identified two groups relative to this feature: with rounded depressions or with irregular lines, but no species with smooth rapheless valves.

According to molecular data, *P. laevis* sp. nov. belongs to a clade that also includes strains of *P. frequentissimum* and *P. naradoense*. Strains of *P. laevis* sp. nov. and *P. frequentissimum* group together, which is interesting since morphologically these two species are clearly differentiated. Strains D06_138, D06_139 and D06_117b have been identified as *P. frequentissimum* and illustrated by Jahn et al. [10] (figs 192–239); however, these strains are morphologically different from the original material of *P. frequentissimum* as illustrated by Wetzel et al. [8] (figs 251–282, 344–353–353) and are in fact more similar to *P. laevis* sp. nov., which aligns with these strains grouping together on the phylogenetic tree. It is likely that our strains and strains D06_138, D06_139 and D06_117b represent the same species, *P. laevis* sp. nov. Strains TCC615 and LCR_S_2_1_1 that are also included in this subclade unfortunately have not been illustrated and thus require further investigation.

### *Pseudoplanothidium* gen. nov.

This genus morphologically resembles *Planothidium* s.str. as well as *Paraplanothidium* gen. nov. but is separated from these genera based on the lack a horseshoe-shaped area in the central part of the rapheless valve. Other differentiating features of *Pseudoplanothidium* gen. nov. include multiseriate striae on both valves that consist of up to 6 rows of areolae each and terminal raphe fissures that are bent to one side and extended onto the mantle.

Two new species are described in this study, with *Pseudoplanothidium foliiformis* sp. nov. designated as the type species of the new genus. On the phylogenetic tree, both new species of *Pseudoplanothidium* belong to a small clade that also includes the strain of *P. suncheonmanense* [10]; all three species form distinct branches within the clade.

*Ps. foliiformis* sp. nov. is quite similar to *Planothidium delicatulum* and related species, especially *Planothidium lilianeanum* Van de Vijver [9] and *Planothidium schwabei* (Krasske) Lange-Bertalot [29]. A detailed comparison of these species can be found in Table 2. *Ps. foliiformis* sp. nov. can be differentiated from *P. delicatulum* and *P. lilianeanum* by valve shape, as both of these species possess protracted, cuneate valve ends while in *Ps. foliiformis* sp. nov. they are rounded or slightly protracted. *P. delicatulum* has a more pronounced central area and more radiate striae on the raphe valve than the new species. *P. lilianeanum* can be differentiated by striae that consist of 3 rows of areolae each (vs. 4–5 rows per stria in *Ps. foliiformis* sp. nov.), a distinctly lanceolate axial area on the rapheless valve and the ultrastructure of the raphe. Both *P. lilianeanum* and *P. delicatulum* also have distinct ornamentation on the rapheless valve [9], while in *Ps. foliiformis* sp. nov. it is mostly unornamented. Another species that resembles *Ps. foliiformis* sp. nov., *P. schwabei*, has not been studied in SEM, so the ultrastructure of this species has not been described; however, the valves of *P. schwabei* are significantly larger than those of *Ps. foliiformis* sp. nov. (length 12.7–20.0 μm in *P. schwabei* vs. 5.7–13.5 μm in *Ps. foliiformis* sp. nov., width 6.7–9.3 μm in *P. schwabei* vs. 3.9–6.9 μm in *Ps. foliiformis* sp. nov.) [29]. The striae on the raphe valve of *P. schwabei* are also wider and more radiate than in the new species. A detailed examination of the ultrastructure of *P. schwabei* would undoubtedly help differentiate it from our new species; however, even now we are fairly confident that these taxa are not conspecific.

**Table 2:** Comparison of *Pseudoplanothidium foliiformis* sp. nov. and related species.

	*Pseudoplanothidium foliiformis* sp. nov.	*P. delicatulum*	*P. lilianeanum*	*P. schwabei*
**Valve shape**	wide lanceolate	elliptic	ellliptic to elliptic-lanceolate	wide lanceolate
**Valve apices**	rounded or very slightly protracted	cuneate-rostrate	cuneate-rostrate	rounded or very slightly protracted
**Valve length, μm**	5.7–13.5	18.0	11.0–18.0	12.7–20.0
**Valve width, μm**	3.9–6.9	8.5	5.0–6.0	6.7–9.3
**Raphe valve**				
**Axial area**	narrow linear	narrow, slightly widened in the centre	narrow linear	narrow linear
**Central area**	mostly absent or small and round	small, rectangular	rounded to rectangular	small, irregular
**Striae pattern**	parallel at the centre, strongly radiate towards the ends	radiate	strongly radiate	radiate
**Rows of areolae in a stria**	4–5 near valve margin, 1–3 near axial area	4–5 near valve margin, 1–3 near axial area	3, all rows equal size	N/A
**Striae density in 10 μm**	13–18	13	16–18	13–15
**Rapheless valve**				
**Axial area**	narrow linear	narrow linear	relatively broad, lanceolate	narrow linear
**Central area**	mostly absent, 2 central striae spaced wider on one side	absent	absent to small asymmetrical	absent
**Ornamentation**	axial area may be slightly depressed	depressed lines between striae and on the axial area	round and elliptical small depressions along the axial area	N/A
**Striae pattern**	parallel at the centre, strongly radiate towards the ends	parallel at the centre, weakly radiate towards the ends	asymmetrical, more radiate on one side of the valve	parallel at the centre, weakly radiate towards the ends
**Rows of areolae on a stria**	4–5	4–5	3, areolae in the middle row smaller	N/A
**Striae density in 10 μm**	14–17	14	16–18	14–15
**Source**	this study	[9]	[9]	[29]

*Pseudoplanothidium minutum* sp. nov. is similar to several established small-celled *Planothidium* s.l. species, the most similar being *Planothidium granum* (Hohn et Hellerman) Lange-Bertalot and *Planothidium minutissimum* (Krasske) Lange-Bertalot. *P. minutissimum* can be distinguished from the new species by protracted valve ends, triangular hyaline area on one side of the rapheless valve and the narrowly lanceolate axial area on the rapheless valve [6]. The striae density in *P. minutissimum* is also lower than in *Ps. minutum* sp. nov. (14–16 in 10 μm vs. 16–19 in 10 μm, respectively). *P. granum* differentiates from the new species by the number of rows of areolae per stria (3–5 vs. 2–3 in *Ps. minutum* sp. nov.) [6]. Also the rapheless valve in *P. granum* is distinctly ornamented and all the striae on the rapheless valve are spaced evenly, while in *Ps. minutum* sp. nov. the central two striae are spaced wider than the rest. Other small-celled *Planothidium* s.l. species, such as *P. suncheonmanense* and *P. pumilum* Bąk et Lange-Bertalot, resemble *Ps. minutum* sp. nov. at first glance, however, they can be easily distinguished from the new species by valve outline.

Molecular data shows that both newly described genera are closely related to *Planothidium* s. str., while being clearly separate entities. *Paraplanothidium* gen. nov. forms a clade that is sister to *Planothidium* s. str., while *Pseudoplanothidium* gen. nov. is removed a bit further from *Planothidium* s. str. It has been shown previously [30] that *Planothidium* is included in the family Achnanthidiaceae D.G. Mann together with other monoraphid genera such as *Achnanthidium*, *Psammothidium* and *Pauliella* Round et Basson, thus the new genera also belong to the Achnanthidiaceae.

### Genetic variability in *Planothidium* s.l.

Previous studies of genetic distances in different groups of diatoms have shown that the divergence in 18S and *rbc*L sequences can vary, and that closely related species sometimes exhibit quite low genetic differences. For example, species of *Sellaphora* Mereschkowsky studied by Vanormelingen et al. showed differences of 0.32–0.40% in *rbc*L between the two most closely related species [31]; the species separation was, in this case, confirmed by mating experiments. Morphologically the studied species of the *S. auldreekie* D.G. Mann & S.M. McDonald complex were extremely similar, and correct identification of them using only morphology was fairly problematic [31]. For representatives of the genus *Pinnularia* Ehrenberg, the most variable marker was in fact the nuclear encoded 28S gene [32]; the maximum sequence divergence between strains of *P. borealis* Ehrenberg was 18.8% for 28S and only 3.5% for *rbc*L. This study showed several distinct lineages in the *P. borealis* complex; the two most closely related lineages differed from each other by 1.4–1.9% in 28S and 0.7–1.0% in *rbc*L. Morphological analysis did not reveal any features that could be used to reliably distinguish between the lineages of *P. borealis* [32]. An interesting case was studied recently in *Humidophila* species [33]: genetic distance between species was generally large, with 1.0–7.4% variation in V4 and 0.9–4.9% variation in *rbc*L, but, additionally, some *Humidophila* strains exhibited genetic differentiation without any consistent morphological differences. In other strains, two distinct morphotypes could be distinguished that were identical genetically [33].

In the group of monoraphid diatoms, a similar study was carried out with the *Achnanthidium minutissimum* (Kützing) Czarnecki complex [34]: 12 lineages were revealed by the phylogenetic analysis, one of which was decribed as a separate species. It was shown that the 18S genetic marker does not have a species level resolution in the *A. minutissimum* complex; *rbc*L was the most variable marker for this group, with strains differing by 0.0–4.4% (compared to 0.0–1.7% in 28S and 0.0–4.2% in 18S). The recovered lineages corresponded to two main morphodemes that could be confidently distinguished with the use of LM and SEM; detailed analysis of one morphodeme showed small but consistent morphological differences between different strains [34]. All these studies show that small (less than 1.0%) genetic distance in commonly used genetic markers 18S and *rbc*L is fairly often seen between closely related diatom species, and it is not always possible to find morphological features that correspond to the results of molecular analysis.

We have examined the genetic distances between our strains of *Planothidium* s.l. and strains of other previously described species. Overall, strains that we have assigned to definite species exhibit considerable genetic variation from other taxa. The complete tables of genetic distances between examined strains can be found in supplementary materials (S1 Table and S2 Table).

The two species with a sinus described earlier from Kamchatka, *P. piipii* and *P. novograblenovii*, are quite closely related to each other according to the phylogenetic tree (Fig. 1); the genetic distance between these two species is 0% in V4 18S rDNA and 0.5–0.7% in *rbc*L. Morphologically, these two species are quite well differentiated by valve shape, sinus shape and striae structure [35]. Both *P. piipii* and *P. novograblenovii* are well separated from other known species by the two used markers: sequence differences between *P. piipii* and the rest of sinus–bearing *Planothidium* taxa are 2.0–3.0% in V4 18S rDNA, 1.0–3.2% in *rbc*L; between *P. novograblenovii* and other *Planothidium* s.str. – 2.0–3.0% in V4 18S rDNA, 0.5–2.6% in *rbc*L.

Among the cavum-bearing taxa, while *P. tyushovii* and *P. dyakonovii* are very similar morphologically, they are separated genetically, differences being 0.3% in V4 18S rDNA and 1.0% in *rbc*L. Another similar species from our samples, *P. laevis* sp. nov., is differentiated from *P. tyushovii* by 2.6–2.9% in V4 18S rDNA and 2.6% in *rbc*L, from *P. dyakonovii* – 2.2–2.5% in V4 18S rDNA, 2.6% in *rbc*L. This supports our conclusion that these are three separate species. The variation between these species and other taxa included in Clade 2 (cavum-bearing species) is in most cases significant. Maximum genetic distance between other species and our taxa stands at 3.2% in V4 18S rDNA, 2.7% in *rbc*L for *P. laevis*; 2.5% in V4 18S rDNA, 2.7% in *rbc*L for *P. dyakonovii*; 2.9% in V4 18S rDNA, 2.7% in *rbc*L for *P. tyushovii*. There are, however, strains of *P. frequentissimum* that are genetically identical to *P. laevis* sp. nov. in one of the markers (strains D06_177b, D06_139, D06_138); as was mentioned earlier, these strains identified as *P. frequentissimum* are in fact morphologically closer to *P. laevis* sp. nov. and probably represent the same species, and the genetic distance of 0% points to this as well. *P. tyushovii* is genetically closest to *P. victori*, with some strains being identical too; this may also be a question of correct identification of *P. victori* strains. Some strains of *P. victori* are identical to *P. dyakonovii* in V4 18S rDNA, but are separated by *rbc*L (difference 0.9–1.0%).

It is worth noting that strains of *P. lanceolatum*, *P. frequentissimum* and *P. victori* exhibit high variation in both V4 18S rDNA (0.2–4.0%, 0–2.5% and 0–0.7% respectively) and *rbc*L (0–2.9%, 0–2.9% and 0–0.9% respectively). Together with morphological variability in these species, it points to the presence of cryptic diversity in these complicated species complexes. Phylogenetic analysis also demonstrates that there are several lineages present in these complexes (Fig 1), which probably represent separate species, however, morphological differences between these lineages are not always apparent. This issue requires further investigation with the use of morphological and molecular analysis, possibly considering ecological preferences as well. Other molecular markers may also prove useful in differentiating between species, but currently fewer sequences of those are available for diatom strains, since 18S V4 and *rbc*L are the most widely used ones; establishing a database of other genetic sequences can be a first step in exploring this direction.

The last two species described as new in this study, *P. foliiformis* sp. nov. and *P. minutum* sp. nov., are well separated from each other: difference between these species is 0.7% in V4 18S rDNA and 5.8–6.0% in *rbc*L. Morphologically they are distinct as well. From other species in this clade, *P. foliiformis* sp. nov. differs by 0.2–3.0% in V4 18S rDNA and 1.4–6.5% in *rbc*L; *P. minutum* sp. nov. – by 1.0–2.7% in V4 18S rDNA and 4.1–5.3% in *rbc*L.

Thus, the studied species of *Planothidium* s.l. are in general quite well separated from each other by genetic distances in at least one of the two used genetic markers, with *rbc*L being the more variable marker in most cases. The new species described from Kamchatka [28,35, this study] are clearly differentiated from strains of other species and from each other, supporting their description as independent taxa. Widely distributed species such as *P. lanceolatum*, *P. frequentissimum* and *P. victori* exhibit high genetic variability in both markers, which, together with their morphological diversity, probably means that these are in fact species complexes, the taxonomic composition of which requires further investigation.

## Supporting information

S1 Table Percent divergence (p-distance) matrix of 56 *Planothidium* s.l. strains on the basis of the partial 18S rRNA gene including the V4 barcoding subregion. (XLSX)

S2 Table Percent divergence (p-distance) matrix of 56 *Planothidium* s.l. strains on the basis of the *rbc*L gene. (XLSX)
